# Research Progress with Membrane Shielding Materials for Electromagnetic/Radiation Contamination

**DOI:** 10.3390/membranes13030315

**Published:** 2023-03-09

**Authors:** Hengtong Zhang, Shudong Lin

**Affiliations:** 1 Guangzhou Institute of Chemistry, Chinese Academy of Sciences, Guangzhou 510650, China; 2 University of Chinese Academy of Sciences, Beijing 100049, China

**Keywords:** electromagnetic radiation, radiation, membrane shielding materials, shielding mechanisms, composite materials

## Abstract

As technology develops at a rapid pace, electromagnetic and radiation pollution have become significant issues. These forms of pollution can cause many important environmental issues. If they are not properly managed and addressed, they will be everywhere in the global biosphere, and they will have devastating impacts on human health. In addition to minimizing sources of electromagnetic radiation, the development of lightweight composite shielding materials to address interference from radiation has become an important area of research. A suitable shielding material can effectively reduce the harm caused by electromagnetic interference/radiation. However, membrane shielding materials with general functions cannot effectively exert their shielding performance in all fields, and membrane shielding materials used in different fields must have specific functions under their use conditions. The aim of this review was to provide a comprehensive review of these issues. Firstly, the causes of electromagnetic/radiation pollution were briefly introduced and comprehensively identified and analyzed. Secondly, the strategic solutions offered by membrane shielding materials to address electromagnetic/radiation problems were discussed. Then, the design concept, technical innovation, and related mechanisms of the existing membrane shielding materials were expounded, the treatment methods adopted by scholars to study the environment and performance change laws were introduced, and the main difficulties encountered in this area of research were summarized. Finally, on the basis of a comprehensive analysis of the protection provided by membrane shielding materials against electromagnetic/radiation pollution, the action mechanism of membrane shielding materials was expounded in detail, and the research progress, structural design and performance characterization techniques for these materials were summarized. In addition, the future challenges were prospected. This review will help universities, research institutes, as well as scientific and technological enterprises engaged in related fields to fully understand the design concept and research progress of electromagnetic/radiation-contaminated membrane shielding materials. In addition, it is hoped that this review will facilitate efforts to accelerate the research and development of membrane shielding materials and offer potential applications in areas such as electronics, nuclear medicine, agriculture, and other areas of industry.

## 1. Introduction

**Figure 1:**
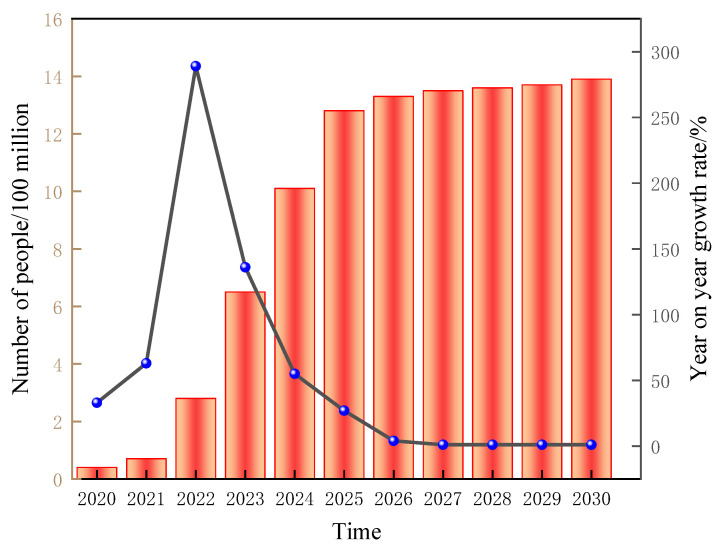
Forecasted numbers of 5G subscribers in China from 2020 to 2030 [1].

**Figure 2:**
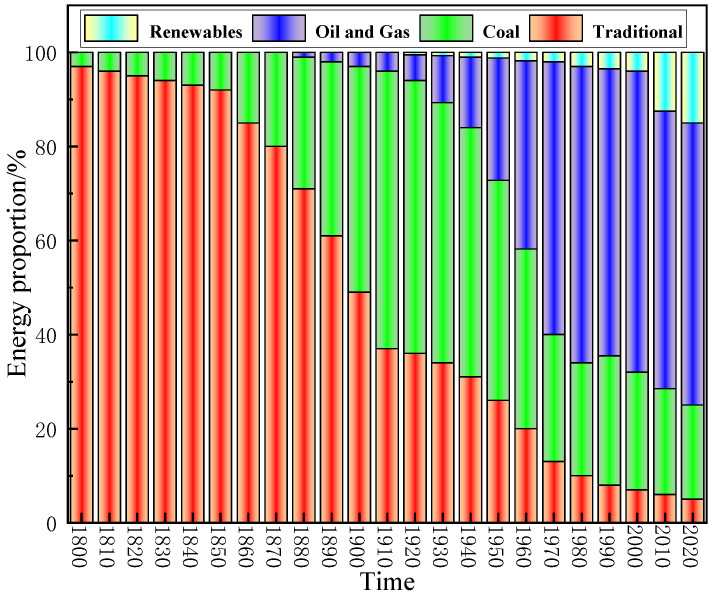
Share of global energy consumed and the change in global primary energy consumption [7].

With the advent of the 5G era, the number of electronic devices has grown exponentially (Figure 1) [1]. However, mobile phones, computers, and radar systems generate electromagnetic pollution, which can seriously interfere with people’s lives and the use of other electronic products and can even pose serious threats to human health while also disrupting the normal operation of devices [2,3,4,5,6]. In addition, as governments seek to find reliable energy sources, the application of nuclear energy is becoming increasingly common (Figure 2) [7]. The radiation generated during the operation of high-end scientific and technological equipment causes various forms of radiation pollution such as medical radiation [8], nuclear reactor radiation [9], scientific research radiation [10] and industrial radiation [11], which are harmful to human health and the environment similar to water pollution and air pollution in modern life. Therefore, it is imperative to find a reasonable and effective method to solve the above problems [12,13,14,15,16]. 

The use of membrane shielding material is a very effective electromagnetic/radiation protection method, which can reduce the radiation by reflection or absorption, especially when the distance and time are limited [17,18,19]. In order to obtain a good shielding effect, appropriate shielding materials should be selected that are appropriate for the given application. Therefore, it is necessary to study the performance characteristics of membrane shielding materials with potential electromagnetic/radiation pollution. In order to handle the environmental pollution caused by electromagnetic/radiation, different membrane shielding materials have been produced to protect human beings and their environment from the destructive effects of electromagnetic/radiation [20,21,22]. When looking for suitable membrane shielding materials, the weight, space, and cost of membrane shielding materials are the primary problems faced by researchers. More importantly, lightweight, non-toxic, and flexible membrane shielding materials with robust mechanical properties and good shielding effects are the common goal pursued by researchers. 

For many years, researchers have studied numerous kinds of membrane shielding materials to deal with radiation pollution caused by electromagnetic/radiation, such as metal-based [23,24,25], polymer-based [26,27,28], concrete-based [9,29,30], lead-based [31,32,33], boron-based [34] materials, as well as other examples of widely used membrane shielding materials. However, with the rapid pace of technological development, the above-mentioned membrane shielding materials can no longer meet the problem of electromagnetic/radiation pollution caused by many types of modern technological devices. Therefore, more researchers have devoted themselves to the exploration of membrane shielding materials to protect humans and their environment, and these efforts have expanded to the preparation of many new membrane-based shielding materials, such as the use of 3D printing design [35,36,37,38], as well as the development of MXene-based [39,40,41,42], carbon-based [43,44,45,46], iron-based [47,48,49,50], cellulose-based [51,52,53,54], and lead-free materials [55,56,57,58,59,60]. The traditional electromagnetic/radiation shielding method was to directly blend conductive fillers to improve the shielding performance [61], especially in the field of electromagnetic radiation shielding. However, it was not easy for the fillers to form an effective continuous network, which made it difficult for electrons to pass through the polymer matrix and hindered efforts to improve the effectiveness of the membrane shielding materials [62]. In addition, commonly used radiation shielding materials are mostly concrete and metals (such as lead, tungsten, iron, etc.) [23,24,25,29,30]. However, concrete has some disadvantages, such as large volume, difficult movement, and poor compressive capacity [9,29,30]. Meanwhile, boron-containing stainless steel has a higher density [34]. Common heavy metals are often toxic, heavy in weight, have a poor melting point, low mechanical strength, and offer poor shielding performance against neutrons [31,32,33]. Fortunately, with the in-depth understanding of the mechanism of membrane shielding and extensive research on the raw materials and preparation technology of membrane shielding materials, efforts were underway to address the above problems. 

With the rapid development of electronics, components and nuclear power sources, comprehensive strategies, and to alleviate and control electromagnetic/radiation pollution have been put forward, and composite membrane shielding materials have quickly gained the attention of researchers. Compared with traditional concrete and heavy metal shielding materials, composite polymer-based materials in which a polymer comprises the matrix and nanomaterials are introduced as fillers that have the advantages of easy molding, light in weight, relatively inexpensive, etc., and thus these materials are promising candidates for applications in aerospace, nuclear power plants, and medical devices [63,64,65]. Most polymer substrates are polymers, such as polyurethane [66,67] and nanofibers [54,68,69,70]. Nanofillers include composite shielding materials such as metal-based materials [71,72], MXene-based materials [73,74,75,76], carbon-based materials [77,78,79,80], and so forth. They have different forms so that they can are suitable for different fields. The combination of a nanofiller and polymer matrix can obtain excellent shielding performance and various unique functions [81]. Therefore, the effective strategy to enhance the electromagnetic interference/radiation shielding performance and meet the actual needs was to construct a hybrid system comprised of a single material combined with multiple materials, thereby integrating the desirable characteristics of different materials and expanding their applicability.

However, portable communication devices such as wearable electronic products and head-mounted sensors are controlled by wireless networks, so their internal electronic components and complex circuits will inevitably produce a large amount of electromagnetic radiation, which will greatly affect the normal operation of high-end precision electronic component systems and human health [12,13,14,15,16]. It was found that [82,83,84] great progress had been made in the preparation of transparent conductive films by using both AgNW and MXene, which can eliminate the influence of electromagnetic pollution on human health, but the complex preparation process and the use of chemical reagents greatly limits their large-scale application. In particular, the transparent conductive films prepared based on polymers are easy to cause internal damage to polymers during the preparation process, which is the biggest obstacle to their further application [83,84]. With the deepening of research, the self-repairing ability was introduced into the polymer matrix by imitating the self-repairing characteristics of cells and tissues to realize the cooperative repair of structure and function, effectively solve the vulnerability problem of polymers, reduce the harm caused by radiation leakage, prolong the service life, and maintain the functional stability of devices [84,85]. The summary of this important subject provides an important theoretical basis for the development of shielding materials with a number of desirable characteristics, such as multifunctional applications, flexibility, low density, low cost, high transparency, and rather light weight.

**Figure 3:**
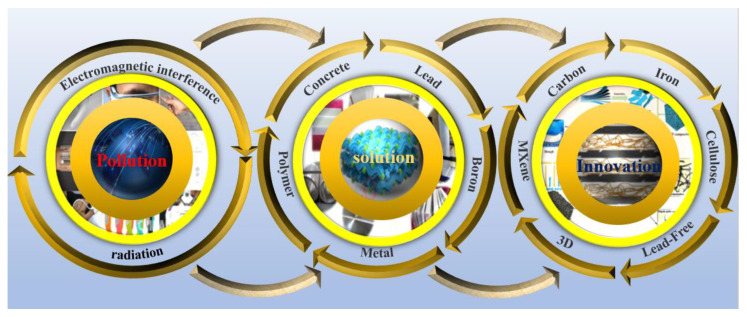
Technical development of membrane shielding materials to handle electromagnetic/radiation pollution.

In this paper, the membrane shielding materials that have been developed to address various forms of electromagnetic/radiation were comprehensively analyzed and discussed (Figure 3). Membrane shielding materials used in different fields have special targeting functions. At the same time, the shielding performance of membrane materials was closely related to the structure and composition of the materials, which were determined by the preparation methods leading to these materials. Therefore, these membrane materials have obtained excellent properties such as thermal stability, flexibility, being light in weight, and exhibiting light transmittance through special modification and preparation methods, and they are suitable for use in specific fields. Based on these considerations, this paper summarizes the research progress of traditional and new membrane shielding materials, puts forward strategic solutions, and expounds the different construction strategies for membrane shielding materials. Finally, the shielding mechanism and structural design principles of shielding materials were deeply analyzed, and the future development prospects and directions for the membrane shielding materials industry were put forward. We believe that this brief review will provide valuable insight regarding the current research status of membrane shielding materials and will help to elucidate the possible research directions for solving the bottleneck in the field of electromagnetic/radiation-contaminated membrane shielding materials. Therefore, we believe that this work will provide inspiration and be a valuable reference tool for those pursuing the field of shielding materials research. 

## 2. Electromagnetic and Radiation Pollution

**Figure 4:**
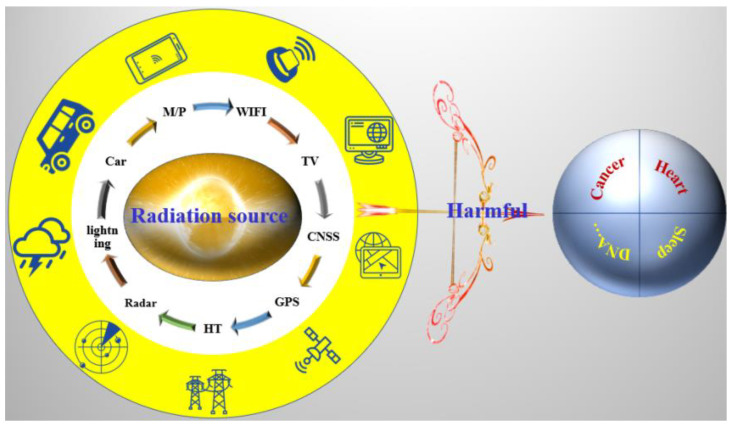
Sources and hazards of electromagnetic pollution.

With the rapid development of electronic technology, the widespread application of high-end precision electronic components and 5G communication systems in aerospace, military engineering, electrical electronics, wireless computers, mobile phones, wearable smart devices, and other fields has greatly enriched our daily lives and changed our lifestyles [74,82]. In recent years, with the rapid growth of electronic communication equipment, the use of electronic products by the eight billion people across the world has increased exponentially. The resulting electromagnetic pollution problem was very prominent, which not only affected the normal work of high-end precision electronic components but also posed a significant threat to human health [82], as shown in Figure 4. According to research reports, economic loss due to electronic equipment failures caused by electromagnetic interference around the world is as high as $500 million USD every year [83]. At the same time, if people all over the world were exposed to radiation for prolonged durations, the health hazards would inevitably become overwhelming. For example, electromagnetic waves can interfere with aircraft navigation [85], cause damage to electronic equipment, and lead to information leakage [84]. In addition, electromagnetic radiation interferes with the normal operation of equipment, causing electronic equipment failure, which leads to serious losses in military and civil applications. Besides, electromagnetic waves can affect the human body, causing different degrees of damage to various organs and tissues [86]. Recent studies have pointed out that these forms of radiation can lead to depression, suicidal tendencies, children’s ADHD, and neuropsychiatric disorders, as well as abnormal births [86]. More importantly, with the development of 5G technology in recent years, people who are often accompanied by mobile phones and computers have become increasingly worried about the health implications of electromagnetic radiation. Compared with the traditional 4G network, which mainly works at around 2.4 GHz, the emerging 5G (6 GHz) network operates at a higher frequency, so it will produce higher energy electromagnetic radiation, which will cause great harm to people’s health and the operation of electronic equipment [87]. In order to protect human health and ensure the normal operation of precision electronic equipment, there is an urgent need for efficient electromagnetic interference membrane shielding materials to eliminate electromagnetic radiation. New electromagnetic interference membrane shielding materials should be light weight, inexpensive, porous, highly efficient, have high thermal conductivity, have wide absorption bands, and offer controllable comprehensive performance. 

**Figure 5:**
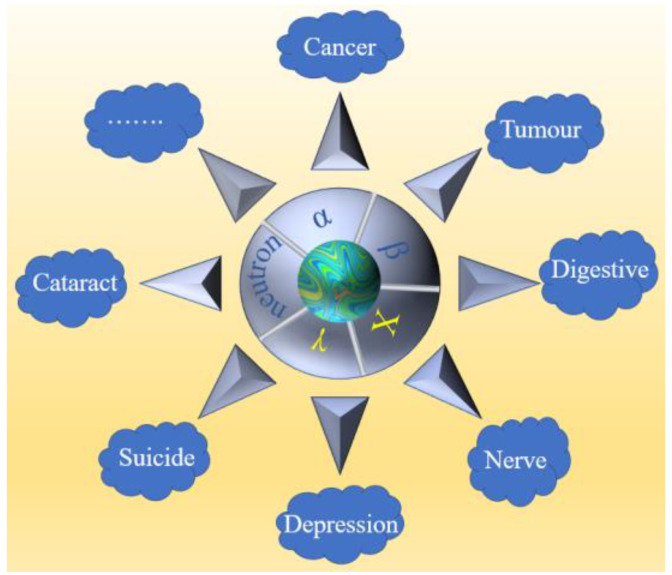
Sources of radiation pollution and their potential health hazards.

With the rapid development of the nuclear energy industry, α, β, x, γ, neutrons and other rays were widely used in medical detection, aerospace, nuclear submarines, nuclear power generation, nondestructive testing, the military, as well as in agriculture and industry [88,89,90]. Although the application of radiation plays a great role in promoting human development and is becoming increasingly important, the existing radiation hazards can not be ignored, as shown in Figure 5. Alpha rays are comprised of mainly helium nuclei, and some radioactive heavy elements will emit alpha particles through alpha decay, thus becoming photons. Once alpha particles are inhaled or injected into the human body, they can destroy the cells of internal organs [90]. Beta rays are a type of charged particle that moves at high speed and is released during the radioactive decay of a nuclide. Originating from either man-made or natural sources, beta rays are a more harmful form of radiation than alpha rays, and they can penetrate more deeply into materials and tissues, including skin [91]. X/γ rays have high photon energy as well as strong penetrating capabilities [92], and they can ionize substances, so that they not only damage human tissues and organs [93], but also pollute the environment, and have thus been classified as Class I carcinogens [94]. A neutron is one of the nuclei that make up the nucleus of an atom. Neutron radiation has a strong penetrating power, and it is more dangerous to the human body than the same dose of X/γ rays, which is 2~14 times that of X/γ rays, and it is also included in the list of Class I carcinogens [95]. After the human body is exposed to radiation, the digestive system and male gonads will become seriously damaged, potentially inducing the formation of tumors, which will easily lead to early death. At the same time, the damaged body was susceptible to severe infection. Therefore, providing effective protection against radiation has a critical role in protecting human health and environmental safety [88,89,90,91,92,93,94,95]. In order to minimize the risk of potential adverse effects arising from excessive radiation exposure, appropriate and effective radiation shielding materials must be utilized in all facilities with radiation to reduce the radiation damage of the target site, especially for the health protection of operators. Therefore, there is an urgent need for a new type of efficient, convenient, nontoxic, and more environmentally friendly membrane shielding material to provide protection against radiation. 

## 3. Comprehensive Strategies and Solutions to Mitigate and Control Electromagnetic/Radiation Contamination

### 3.1. Metal-Based Membrane Shielding Materials

**Figure 6:**
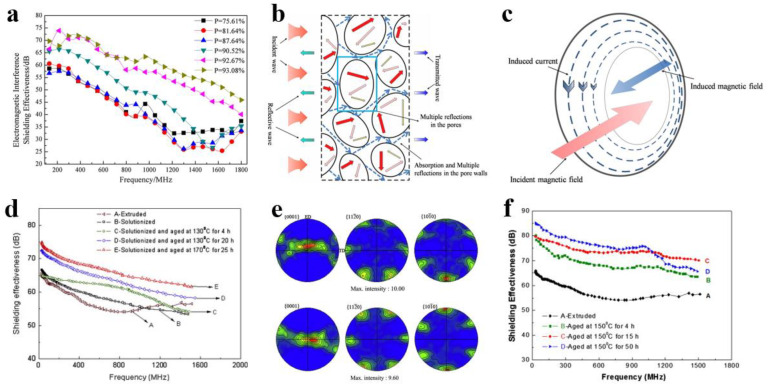
(**a**) Shielding effect of aluminum foam membrane [98]. Copyright 2014, Elsevier. (**b**) Transmission effect of electromagnetic wave in foamed aluminum [98]. Copyright 2014, Elsevier. (**c**) Electromagnetic induction principle of foamed aluminum [99]. Copyright 2015, Elsevier. (**d**) Shielding efficiency of ZK60 alloy membrane [100]. Copyright 2012, Elsevier. (**e**) Electron backscattered diffraction (EBSD) measurement of polar diagram of alloy before and after heat treatment [100]. Copyright 2012, Elsevier. (**f**) Shielding efficiency of ZK60 magnesium alloy membrane [100]. Copyright 2013, Elsevier.

Metal and its alloy materials have excellent conductivity. For example, the conductivity of copper and aluminum can reach 16~17 S/cm, and thus these materials can absorb, reflect, and transmit electromagnetic interference [27]. Therefore, metals and their alloy materials were first used as electromagnetic membrane shielding materials [96,97]. Xu et al. [98] prepared an aluminum foam membrane shielding material by a melt foaming method that has a good shielding effect (25–75 dB) on space plane electromagnetic waves with frequencies in the range of 130–1800 MHz (Figure 6a). The mechanism through which the shielding effect was analyzed, as shown in Figure 6b. With further investigation, researchers found that eddy current loss was an important shielding form [99], and the structure of aluminum foam described in this paper just determines the existence of eddy current loss in aluminum foam, as shown in Figure 6c. Although metal has high electromagnetic shielding effectiveness, it also has some disadvantages, such as high density, poor flexibility, low corrosion resistance, and high processing cost, which limit its applicability [23,24,25,96,97]. Compared with pure metal, magnesium alloy has better electromagnetic shielding performance as well as lower density, and thus it may be considered as a potential electromagnetic interference shielding material [99]. Chen et al. [100] found that the shielding effectiveness of the ZK60 alloy membrane was significantly improved by heat treatment, reaching up to 75 dB (Figure 6d), and it had good mechanical properties. More importantly, through research on the shielding mechanism (Figure 6e), it was found that ZK60 magnesium alloy precipitates a large number of second phases in the supersaturated matrix, which leads to better electrical conductivity, thus enhancing the shielding ability of ZK60 magnesium alloy. To further investigate the shielding effect of magnesium alloy, Chen et al. [101] studied the shielding effect of a ZK60 magnesium alloy membrane under different aging conditions (Figure 6f). The shielding effect was as high as 70 dB, and the tensile strength reached 316 MPa. However, alloying produces an excessive second phase, which reduces the effectiveness of membrane shielding materials. In addition, it has the disadvantages of being prone to corrosion and having insufficient flexibility, and it is easy to form secondary pollution, which greatly limits its applicability. Therefore, it is necessary to develop membrane materials with low density, good mechanical properties, especially excellent electromagnetic interference/radiation to prevent radiation.

**Figure 7:**
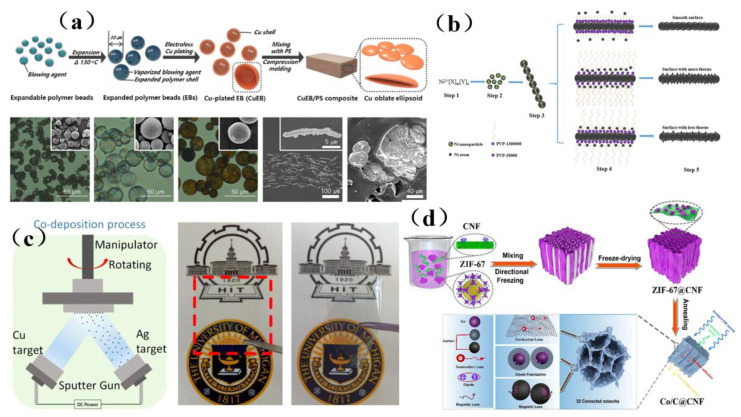
(**a**) Schematic diagram of shielding material comprised of polystyrene composite membrane containing copper oblate ellipsoid particles [103]. Copyright 2017, Elsevier. (**b**) Schematic diagram of the PVP-regulated mechanism for the growth of NiNWs [104]. Copyright 2019, Elsevier. (**c**) Schematic diagram of co-deposition process and transparency comparison of Ag/Cu/PET transparent membrane for electromagnetic interference shielding [105]. Copyright 2019, American Chemical Society. (**d**) Schematic diagram depicting the fabrication of Co/C@CNF aerogel and schematic diagram of the electromagnetic interference (EMI) shielding mechanism [106]. Copyright 2020, Elsevier.

In recent years, researchers have developed many methods to fabricate shielding materials, including the preparation of metal/polymer, metal/fabric, transparent metal membranes, aerogels, and other composite materials [102]. Metal/polymer composites, which combine the excellent electrical conductivity of metal with the excellent mechanical properties of polymers, can overcome the shortcomings of traditional metal shielding materials such as rigidity and high density, while offering a good shielding effect. Seung et al. [103] prepared a highly anisotropic polystyrene composite containing copper oblate ellipsoid particles. Due to the presence of Cu, the conductivity of this composite material was greatly enhanced, and its special layered structure allows electromagnetic waves to be absorbed by this material, which can shield 300 kHz~12 GHz broadband electromagnetic waves with the highest shielding efficiency of 80 dB. The preparation process is shown in Figure 7a. Compared with metal/polymer composite membrane materials, metal/fabric composite membrane materials have the advantages of low filler content, low density, and strong interfacial adhesion, which is conducive to the dispersion of metal fillers in the matrix and the enhancement of interfacial polarization of materials. However, its shielding effectiveness is weaker than that of metal/polymer composite membrane materials at 8.0~12.4 GHz.Yu et al. [104] prepared Ni nanowires (NiNWs) by a hydrazine hydrate reduction method and dispersed NiNWs in polyvinylidene fluoride. When the filler content (mass percentage) was 9%, the shielding effectiveness of the composite could reach 43 dB at 8.2~12.4 GHz. The polyvinylpyrrolidone (PVP)-controlled growth mechanism of NiNWs was shown in Figure 7b. In most cases, a metal plate is used as the substrate, and the overall density of the material decreases with limited space and the fill volume is large, which makes it difficult to meet the shielding requirements and provide the optical transparency needed for many electronic devices. It has been found that a uniform internal distribution as well as a dense film can be obtained when the metal shielding membrane is prepared via magnetron sputtering. Moreover, this material can exhibit multi-functional characteristics such as light transmission and hydrophobicity while also exhibiting a good shielding effect against high-frequency electromagnetic radiation [102]. Wang et al. [105] prepared a Cu-doped Ag thin membrane by magnetron sputtering (Figure 7c), and its optical transmittance was found to reach 96.5%, which suggested that it can be used as the window of a shield. The thickness of the membrane was only 88 nm, which can be connected with some existing processes, and the control was flexible and convenient. The average shielding effectiveness of the membrane against X (8~12 GHz), Ku (12~18 GHz), Ka (18~27 GHz) and K (26.5~40 GHz) radiation reaches 26 dB. In addition, researchers have also found that the need for high-density metal materials can be reduced by establishing three-dimensional porous structures, and the formation of internal holes in metal aerogel materials can enhance the multiple reflection loss of electromagnetic waves in electromagnetic shielding, thus obtaining good shielding performance [102]. Yang et al. [106] assembled a Zeolitic Imidazolate Framework-67 (ZIF-67)@CNF aerogel, where ZIF-67 and CNF were used as building blocks for a new three-dimensional ultra-light Co/C@CNF aerogel (Figure 7d). Its shielding efficiency was 35.1 dB, and it has extremely high absorption characteristics. This good performance was due to the presence of magnetic cobalt nanoparticles that are embedded in the carbon sheet and the three-dimensional interconnection network, which enhances magnetic loss and dielectric loss. This method provides a simple method to prepare ultra-light and ultra-high absorption aerogels with excellent electromagnetic shielding performance.

In summary, the rapid pace of scientific and technological developments has led to the need for better membrane shielding materials, and the traditional metal membrane shielding materials can no longer meet the demand. It is necessary to study metal-based membrane shielding materials in depth. The goal of developing light weight and flexible metal films can be achieved by coating metal particles onto polymer matrices, but it is difficult to combine a polymer interface that lacks polar functional groups with metal particles. The key direction for the development of transparent metal composite films was toward achieving high transparency, stretchability, and low fill volume. The biggest characteristics encountered with metal aerogels were their high shielding effectiveness, positive resilience, and ultra-low density. We predict that the search for solutions to the challenge of combining and uniformly dispersing metal particles with other materials will become a hot issue in the research of metal-based membrane shielding materials in the next few years.

### 3.2. Polymer-Based Membrance Shielding Materials

Although the density, flexibility and corrosion resistance of metal-based membrane shielding materials have been greatly improved, their flexible wear performance is still greatly limited [107]. The use of polymer-based membrane shielding materials can not only mitigate the disadvantages of metal-based materials (such as their high density, susceptibility to corrosion, difficult preparation, and high cost), but also address the challenges encountered with the design of polymer-based materials [28]. In polymer technology, two kinds of materials with different properties can be combined through the use of a mixed filler system so that the resulting composite material has specific properties, which is called a filled polymer-based membrane shielding material [26]. In addition, there is a more advanced class of membrane shielding material, called conductive polymer fiber composites (ECPCs), which are obtained with the use of conducting polymers that have conjugated segments (such as those with as p-π, or π-π conjugation) [108]. What’s more, conductive polymers are light and flexible [109,110], and it was expected that these polymers would offer a new generation of electromagnetic interference membrane shielding materials offering excellent performance.

**Figure 8:**
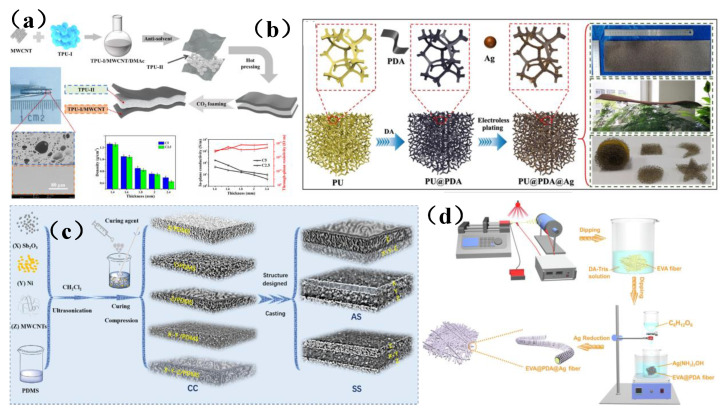
(**a**) Schematic diagram of the preparation process leading to TPU/MWCNTs of its structure and performance [109]. Copyright 2020, Elsevier. (**b**) Schematic diagram of the preparation and performance evaluation of PU@PDA@Ag [111]. Copyright 2020, Elsevier. (**c**) Schematic diagram of the manufacturing process leading to traditional Sb_2_O_3_-Ni-MWCNTs/PDMS composite (CC), asymmetric structure composite (AS) and symmetric structure composite (ss) [107]. Copyright 2022, Elsevier. (**d**) Schematic diagram showing the preparation of EVA@PDA@Ag fiber composites [28]. Copyright 2022, Elsevier.

It is well known that electrical conductivity is one of the key factors affecting the electromagnetic shielding performance of ECPCs [108]. The higher the electrical conductivity, the better the electromagnetic shielding performance. However, traditional ECPCs have some significant shortcomings, such as the strong π-π interactions that occur between conductive fillers with high specific surface area, which increase their tendency to aggregate and thereby make it difficult to obtain a uniform dispersion of fillers in the polymer matrix [28,109,110,111]. In addition, there are many free interfaces between conductive fillers and the polymer matrix, which hinder the continuous transmission of electrons. Therefore, the mechanical strength and shielding capabilities of ECPCs were inevitably weakened. To improve the electromagnetic shielding performance of ECPCs, Song et al. [109] prepared sandwich thermoplastic polyurethane (TPU) foam with adjustable frequency-selective electromagnetic shielding performance using a carbon dioxide intermittent foaming method. Multi-walled carbon nanotubes (MWCNTs) were used as conductive fillers that were selectively distributed on the surface layer, thereby yielding a TPU/MWCNTs composite with foam-selective electromagnetic shielding performance (Figure 8a). The maximum shielding efficiency and absorption rate could reach 53.3 dB and 0.66, respectively, and it showed an interesting and efficient electromagnetic interference frequency selective shielding. Liang et al. [111] modified poly dopamine (PDA) on the surface of polyurethane (PU), and then prepared flexible sponge-like PU@PDA@Ag composites via the in-situ growth of silver (Ag) nanoparticles on the surface of PU (Figure 8b). The research shows that the EMI shielding effectiveness (EMI SE) of PU@PDA@Ag composites was as high as 84 dB, and the absolute shielding effectiveness (SSEt) was as high as 5250 dB cm^2^ g^−1^. Meanwhile, PU@PDA@Ag sponge has low thermal conductivity (52.72 mW/mK), excellent compression elasticity, and piezoresistance. Therefore, PU can be used to construct flexible and highly elastic electromagnetic membrane shielding materials. He et al. [107] used polydimethylsiloxane (PDMS) as a matrix and added multi-walled carbon nanotubes (MWCNTs), nickel (Ni), and antimony trioxide (Sb_2_O_3_) particles as fillers to prepare a PDMS-based electromagnetic membrane shielding composite with various fillers and layered structures (Figure 8c). Among them, symmetric structure (SS) and asymmetric structure (AS) composites can achieve high EMI SE_T_/absorption coefficients of 57.4 dB/0.75 and 55.7 dB/0.80, respectively. This study provides an effective design concept for improving microwave absorption capabilities by using the synergistic effect and a controllable distribution of fillers. Wei et al. [28] used an electrospinning method to prepare a highly cross-linked ethylene-vinyl acetate copolymer (EVA) fiber membrane and then synthesized a poly-dopamine layer on the surface of this film, thus promoting the reduction of Ag nanoparticles on the surface of the EVA@PDA fiber composite, and thereby preparing an ultra-thin and flexible EVA@PDA@Ag fiber composite (Figure 8d). The conductivity of shape-memorized conductive polymer fiber composite (SMCPFC) can reach 2.5 × 10^5^ S/m, and the average EMI SE at 3.94~5.99 GHz was approximately 90 dB. After cyclic stretching, folding, and ultrasonic washing, the conductivity and electromagnetic interference SE remain unchanged. In addition, the SMCPFC exhibits an excellent shape memory effect driven by electric heat, which can block more than 99.99% of electromagnetic waves. The invention of this multifunctional SMCPFC has opened a new avenue for the development of intelligent and functional flexible printed circuit (FPC) electromagnetic shielding membranes.

**Figure 9:**
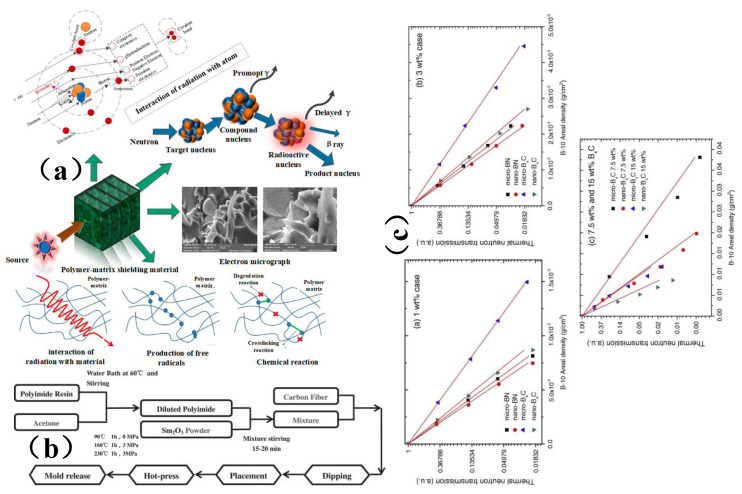
(**a**) A schematic diagram of the interaction between polymer-based nuclear radiation shielding materials and radiation and its effects [112]. Copyright 2021, Frontiers Media S.A. (**b**) A flow chart showing the manufacturing method leading to continuous a carbon fiber-reinforced Sm_2_O_3_/polyimide shielding material [113]. Copyright 2015, Elsevier. (**c**) Thermal neutron transmittance of composite membrane shielding material [114]. Copyright 2014, Elsevier.

A polymer-based nuclear radiation shielding material is an important part of a nuclear power plant [112]. Due to the long-term synergistic effect of nuclear radiation and the thermal effect, its mechanical properties and shielding performance will gradually deteriorate. Its mechanical properties and shielding performance determine whether the device can run safely, which brings hidden dangers to the safe operation of the device [112]. Figure 9a is a schematic diagram of the interaction between a polymer-based nuclear radiation shielding material and radiation and its effect. For polymer-based nuclear radiation shielding materials, polyethylene, resin, and rubber were usually used as the matrix. Good neutron and gamma-ray shielding effects can be achieved via the addition of boron carbide, lead, tungsten, and other reinforcing phase particles to such a matrix [112,113]. Wang et al. [113] prepared a continuous carbon fiber-reinforced Sm_2_O_3_/polyimide γ-ray/neutron shielding material by a hot-pressing method (Figure 9b). This material not only has good shielding performance, but also excellent temperature and pressure resistance, and is thus a promising candidate for use in fusion reactor systems and nuclear waste treatment applications. Kim et al. [114] fused and blended nanoscale boron carbide (B_4_C) and boron nitride (BN) powders with high density polyethylene (HDPE) to prepare a sheet film composite, respectively. The subsequent evaluations revealed that the thermal neutron shielding performance of polymer nanocomposites was more effective than that of micro-composites (Figure 9c), and the mechanical properties were also better. In summary, on the one hand, this study can establish the mapping relationship between the properties and properties of polymer-based membrane shielding materials and radiation and thus provide technical means for the design and development of polymer-based membrane shielding materials. On the other hand, this study can provide a technical basis for the safety evaluation of polymer-based nuclear radiation shielding materials and allow researchers to assess their suitability for long-term use.

### 3.3. Concrete-Based Membrane Shielding Materials

**Figure 10:**
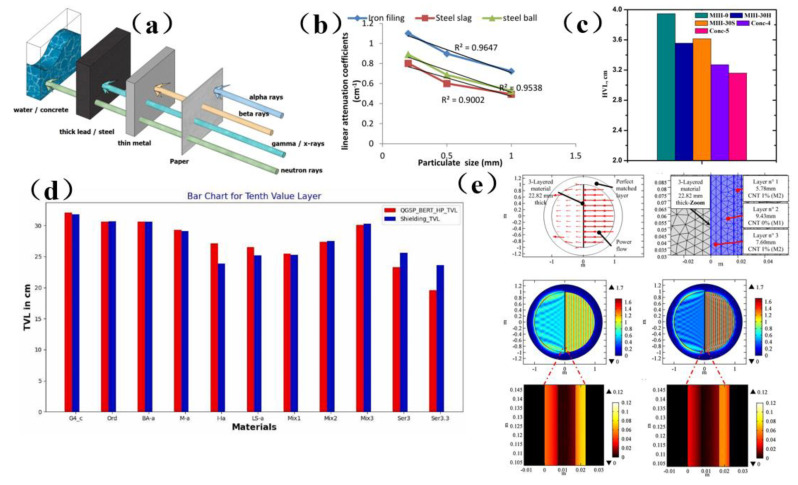
(**a**) Types, penetration behavior, and properties of various types of radiation [115]. Copyright 2022, MDPI. (**b**) Linear attenuation coefficient is a function of filler particle size [117]. Copyright 2014, Trans Tech Publications. (**c**) The half value layer for shielding materials at 0.662 MeV. [118]. Copyright 2022, Elsevier. (**d**) Bar chart for tenth value layer [119]. Copyright 2023, Elsevier. (**e**) Finite element simulation of frequency selective reinforced concrete composite structure [120]. Copyright 2017, Elsevier.

Concrete is non-toxic, fireproof, and readily available from a wide range of sources. It was a very useful material for radiation shielding applications, and it can be used to shield against the radiation hazards of α, β, X, γ, neutrons, and other rays. Its radiation performance was better than that of paper, thin metal, thin lead, steel, and other materials (Figure 10a) [115]. Since 1975, concrete has been used in the construction of nuclear power plants to prevent nuclear waste pollution, and it was often called radiation shielding concrete (RSC) [115,116]. In recent years, the primary approach to attenuate the harm of radiation has been to change the composition of concrete. Omid et al. [116] studied the influence of various water-cement ratios on the shielding characteristics of heavy magnetite concrete. As the water-cement ratio was decreased from 0.7 to 0.4, these parameters for Cs ^137^ −1.333, Co^60^ −1.173, and Co^60^ −0.622 MeV sources decreased by 26.8%, 30.9% and 23.2%, respectively. This kind of heavy magnetite concrete with a low water-cement ratio can shield gamma radiation even with a small thickness, and thus it was a promising candidate for situations requiring concrete shielding materials but where space was limited. Ali et al. [117] found that the shielding effect of concrete mixed with iron filings was the best, and the maximum linear attenuation coefficient achieved with iron filler was 1.102 ± 0.263 ccm ^−1^ (Figure 10b). Al-Ghamdi et al. [118] studied the radiation shielding performance of heavy concrete samples with different concentrations of tungsten oxide. It was found that the use of tungsten oxide increases the density and photon shielding capability of the sample. In the selected energy range, high-density concrete (Conc-5) absorbs gamma photon rays more effectively than low-density samples. The radiation protection efficiency (RPE) of Concr-5 was 99% at 0.122 MeV, which indicated that this kind of concrete can block almost all low-energy incident photons (Figure 10c). Therefore, choosing appropriate materials to shield radiation should be the main consideration in radiation protection design.

In addition, researchers have also used simulation to determine the radiation shielding parameters of any materials and composites using different radiation sources. Paul et al. [119] calculated the shielding characteristics of concrete by the Monte Carlo method and confirmed that the model can be used to determine the radiation shielding parameters of any material and composite material with different radiation sources. (Figure 10d). At the same time, shielding materials in other fields can also be evaluated by this simulation model. Micheli et al. [120] designed a multi-layer structure with the help of particle swarm optimization (PSO) algorithms, and they subsequently analyzed the carbon nanotube reinforced concrete composite material by the numerical finite element method (FEM) (Figure 10e). This technology has a bright future in addressing issues related to electromagnetic interference. In summary, the shielding material of concrete played an excellent role in blocking radiation, but its durability and mechanical properties were poor. Areas requiring further attention include improving the radiation shielding, workability, durability, mechanical properties, crack resistance, impermeability, shrinkage, and other properties of heavy concrete, thereby ensuring that these materials can satisfy the shielding requirements of future devices.

### 3.4. Lead-Based Membrance Shielding Materials

**Figure 11:**
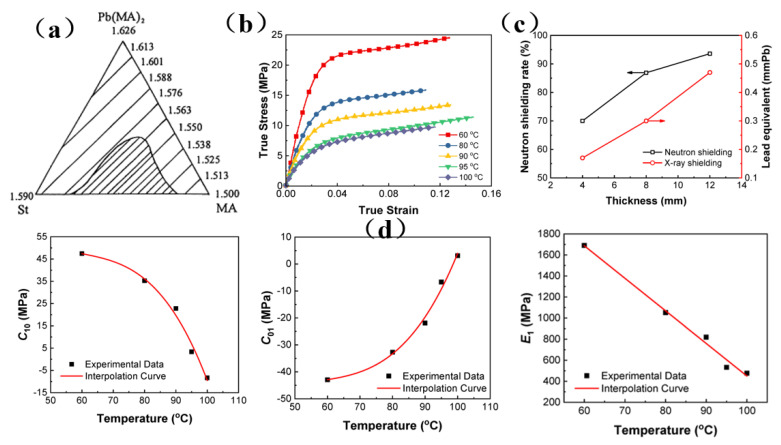
(**a**) Polymer—transparent phase diagram of Pb(MA)_2_/St/MA ternary copolymerization system with the same refractive index line (shaded areas are transparent areas) [121]. Copyright 2000, MDPI. (**b**) The stress—strain curves of Gb/Pb acrylate at various temperatures under the same stretching rate of 1.667 × 10^−4^ s^−1^ [33]. Copyright 2022, MDPI. (**c**) The radiation shielding performance of metallized acrylic polymer was evaluated by lead equivalent thickness [33]. Copyright 2022, MDPI. (**d**) The variation law of model parameters with temperature and interpolation curves. (b, c, d) [33]. Copyright 2022, MDPI.

Because of its low cost, ready availability, and excellent shielding effect, lead has become the first choice for γ-ray shielding materials used in nuclear reactors [31]. However, lead-based materials are toxic and volatile, which leads to lead poisoning reactions in the human body [32]. Therefore, it is necessary to prepare composite materials to reduce the toxicity of lead-based materials. Lin et al. [121] synthesized a lead dimethacrylate compound, and an optical resin containing Pb^2+^ ions was obtained by copolymerization. It was found that the addition of Pb^2+^ to optical resin not only imparts high X-ray absorption and good visible light transmission capabilities (Figure 11a), but also can improve the glass transition temperature, refractive index, and shielding performance of the resin. Zhang et al. [33] successfully synthesized a transparent metallized acrylate-based polymer containing Gd and Pb through the bulk polymerization of organometallic acrylic monomers, and they found that this newly synthesized material had good optical transparency, acceptable thermal stability, good mechanical properties, and excellent shielding properties (Figure 11b,c). According to the modified constitutive model, it was concluded that the Maxwell viscoelastic unit played a decisive role in influencing the response of materials to loads (Figure 11d). Therefore, the established constitutive model can accurately describe the nonlinear viscoelastic tensile response of metallized polyacrylates containing Gd and Pb under quasi-static loading. In summary, lead-based composite materials will likely be a key area of research for the future development of lead-containing shielding materials.

### 3.5. Boron-Based Membrance Shielding Materials

**Figure 12:**
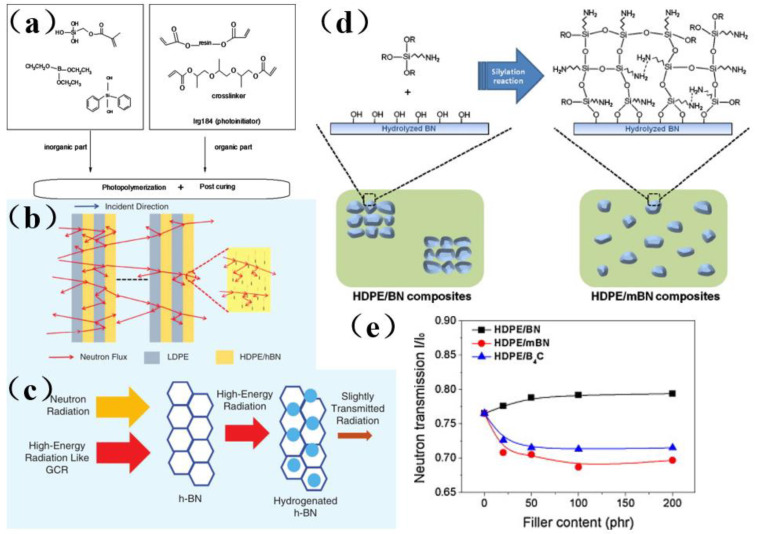
(**a**) Preparation of a hybrid coating containing boron [122]. Copyright 2021, IEEE. (**b**) Schematic diagram of radiation protection effect of hydrogen boron nitride and hydrogenated hydrogen boron nitride [123]. Copyright 2017, Elsevier. (**c**) Multilayer PE/hBN composites and their neutron shielding properties [124]. Copyright 2017, Elsevier. (**d**) Schematic diagram of surface modification of BN with trialkoxysilane [125] Copyright 2014, Elsevier. (**e**) The relationship between neutron transmission coefficient of HDPE/BN, HDPE/mBN and HDPE/B_4_C composites and filler content. d, e. [125] Copyright 2014, Elsevier.

It was known that boron could absorb neutrons [122,123,124,125,126,127,128,129,130,131,132,133,134,135,136,137,138,139,140,141,142,143,144,145,146,147,148,149,150,151,152,153,154,155]. If a boron-containing coating material has sufficient thickness, most charged neutron particles will be absorbed, and boron-containing materials can prevent neutron migration. Because of this characteristic, boron can be used to simplify the preparation process and lower the cost of the resultant material [122]. Mülazim et al. [122] prepared an ultraviolet−cured boron-containing hybrid coating from a mixture of acrylic bisphenol A epoxy resin, methacryloxymethyl triethoxysilane and boric acid by means of anhydrous sol-gel technology (Figure 12a). This coating has a good shielding effect on neutron radiation, and improves the hardness, chemical resistance, wear resistance, and adhesion of boron-containing hybrid materials. It can be used in research and test reactors to shield neutron rays. Considering that boron is widely regarded to be as the best material for use in shielding materials, many recent studies have focused on the hydrogen absorption performance of boron nitride and its radiation shielding effect (Figure 12b) [123]. In addition, the structure of composite material was also very important to improve the radiation shielding effect, especially because the incident radiation of multilayer structure was more easily scattered and absorbed by the shielding layer. (Figure 12c) [123,124]. Functionalized boron nitride can be used in the manufacture of composite materials. Treated silane boron nitride (mBN) has been used to prepare high density polyethylene (HDPE)/boron nitride composites. Figure 12c shows the reported neutron transmittance performance, which indicates that the developed material has an excellent radiation shielding effect. On the other hand, boron nitride treatment leads to stronger adhesion at the interface between boron nitride and a polymer. Ji et al. [125] prepared HDPE composites with modified boron nitride (mBN) filler that had been functionalized with an organosilane compound (Figure 12d) by a traditional melt extrusion process, and the resultant material exhibited well-dispersed filler particles as well as excellent neutron shielding performance (Figure 12e). The HDPE/mBN composites prepared in this study surprisingly showed ultra-high neutron attenuation capabilities over a wide range of filler concentrations. To sum up, in order to obtain high-performance boron-based membrane shielding composites, it was necessary not only for each component (matrix and filler) to have excellent shielding performance but also for the components to have good compatibility and appropriate interfacial properties.

## 4. Novel Electromagnetic/Radiation Shielding Membrane Materials and Technologies

### 4.1. 3D Printing Design of Membrane Shielding Materials

**Figure 13:**
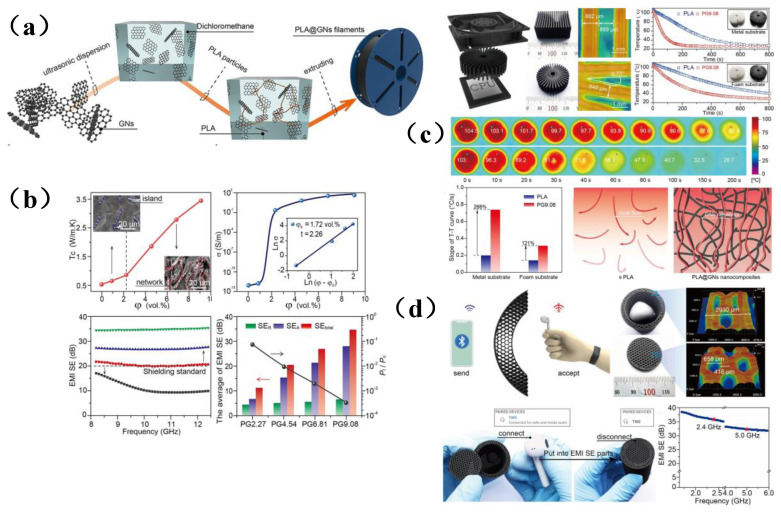
(**a**) Schematic diagram of the preparation process leading to PLA@GNS filament. (**b**) a−b: thermal conductivity and electrical conductivity of PLA@GNS nanocomposites, c: EMI SE characteristics of PLA@GNS nanocomposites as a function of frequency, d: and corresponding electromagnetic parameters and EMWs transmittance. (**c**) a: chemical diagram of heat sink model, b−c: 3D printed digital and super-depth-of-field images of heat sinks, d: heat dissipation curves of pure PLA and PG9.08 filaments prepared via different thermal management environments: metal and foam substrates, e: representative infrared thermal images of heat dissipation behavior of pure PLA and PG9.08 heat sinks on metal substrates, f: corresponding initial heat dissipation rates on metal and foam substrates, g: Schematic diagram of heat dissipation mechanism of pure PLA and PLA@GNs heat sinks. (**d**) Schematic diagram of EMWs shielding module. [127] Copyright 2022, Elsevier.

Although the above solutions have effectively promoted the development of membrane shielding composite materials and have addressed the issues arising from electromagnetic radiation to a certain extent, these materials still have some shortcomings, such as the need for complicated preparation processes, a long production cycle, expensive production equipment, and poor shielding efficiency in special fields. Among the many manufacturing technologies available to generate membrane-based shielding materials, 3D printing technology is one of the latest frontiers in the manufacturing field. It was particularly used to design and prepare complex shapes and structures that are difficult to manufacture by traditional technologies, and this approach has been widely used in various high-tech fields [126]. 3D printing can be controlled by a computer, thus reducing labor costs. In the process of shielding material preparation, it was usually necessary to lay some special materials for continuous layers., such as metal alloy, polylactic acid, graphene, carbon nanotubes, MXene, etc., which increases the cutting and designability of the structure and expands the potential applicability of 3D printing technology in various fields [35]. Due to the unique layer-by-layer stacking method used for this approach, 3D printing shows an exciting prospect. Shi et al. [127] introduced graphene nanosheets (GNs) into a polylactic acid matrix by a solution blending method, and they prepared a promising multifunctional filament (Figure 13a), which endowed the composite filament with ideal thermal conductivity and shielding performance, which reached 3.22 W/m·k and 34.9 dB, respectively, and the shielding efficiency of EWMs energy was 99.97% (Figure 13b). Thereafter, in order to explore the manufacturing potential of potential applications of 3D printing technology, an ideal material with a free structure and excellent performance was constructed. Especially in thermal management, the corresponding initial heat dissipation rate was 266% higher than that of pure radiator. At the same time, the mechanism was explored, mainly because multifunctional filament electron transfer played a key role in heat flow (Figure 13c). In addition, the shielding module obtained via 3D printing has high shielding performance (35.8 dB) under a specific Bluetooth interactive signal (2.4 GHz) (Figure 13d). In general, this innovative research not only enriches the printable materials with customized multifunctional features but is also anticipated to provide a promising route toward the next generation of multifunctional devices with applications in modern electronic engineering and great market competitiveness.

**Figure 14:**
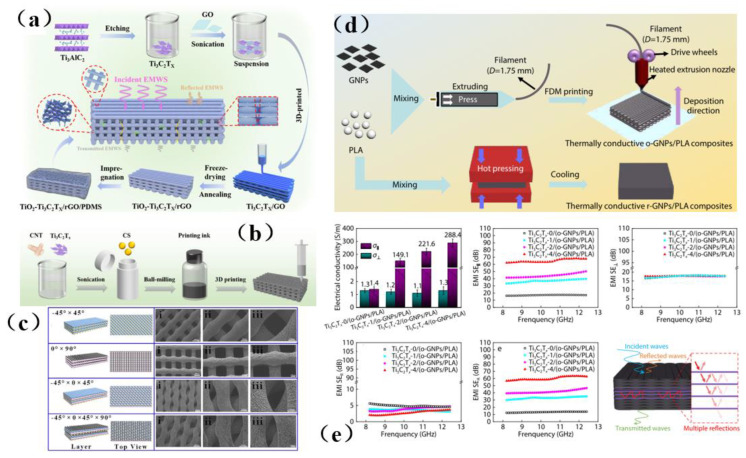
(**a**) Schematic diagram of preparation depicting the Ti_3_C_2_T_x_/rGO/PDMS composite [128]. Copyright 2022, Elsevier. (**b**) Preparation process of CNT/ Ti_3_C_2_T_x_/CS ink and its 3D printing composite carbon nanotubes; CS, chitosan. (**c**) Schematic diagram depicting the preparation of porous 3D printed composites with different structures. b, c [129]. Copyright 2022, Wiley. (**d**) Schematic diagram depicting the preparation of thermal conductive GNPs/PLA composites. (**e**) EMI SE and EMI shielding diagram of Ti_3_C_2_T_X_/(O—GNP/PLA) thermal conductive composite. d, e [126]. Copyright 2022, Chinese Chemical Society and Institute of Chemistry.

3D printing technology can provide a high degree of control over the microporous structure of the resultant material, thus facilitating efforts to achieve the functions and properties required by EMI shielding materials. Liu et al. [128] used 3D printing technology to construct a Ti_3_C_2_T_x_/GO framework with a vertical pore gradient and then cured and annealed the 3D framework with PDMS to prepare a TiO_2_-Ti_3_C_2_T_x_/rGO/PDMS composite with high shielding efficiency (Figure 14a). It was worth noting that this membrane shielding material has a unique multi-layered scale structure. In addition, simulation studies have been employed to investigate the influence of the gradient aperture on the electromagnetic interference of composite materials. Under the synergistic effect of multiple loss mechanisms, the designed composite exhibits a conductivity as high as 173.1 S/m and an excellent EMI SE of 58 dB. The same shielding material also has excellent thermal management performance. The porous structure prepared in this study fully demonstrates the potential of personalized design and customization using 3D printing technology, thus broadening the potential applications of 3D printing technology in the field of electromagnetic/radiation membrane shielding materials. Generally, membrane shielding materials with porous structures can promote the internal reflection and scattering of electromagnetic waves, and often have better shielding effectiveness than non-porous materials. Pei et al. [129] prepared porous CNT/Ti_3_C_2_T_x_/CS composites by a combination of ball milling technology and 3D printing technology (Figure 14b,c). It was found that CNTs and Ti_3_C_2_T_x_ were uniformly dispersed in the polymer matrix, which is conducive to the construction of a conductive network thus improves the electromagnetic shielding performance of 3D printing devices, which can reach 23.5 dB. In addition, melting deposition modeling (FDM) 3D printing technology makes use of the orientation of melt flow during polymer extrusion, which enables the orderly arrangement of fillers as well as the convenient and efficient construction of the filler network. Ma et al. [126] used FDM 3D printing technology to prepare ordered GNPs/PLA sheets with PLA and graphene nanosheets (GNPs) as auxiliary materials. Then, a Ti_3_C_2_T_x_ /(O-GNPs/PLA) composite material with good shielding performance and thermal conductivity was prepared by “layer-by-layer lamination-hot pressing” technology and vacuum-assisted filtration (Figure 14d). Its thermal conductivity and shielding effectiveness were as high as 3.44 W·m^−1^·K^−1^ and 65 dB, which are increased by 1223.1% and 2066.7%, respectively, compared with PLA matrix material (Figure 14e). This work provides a novel and simple way to design and manufacture high thermal conductivity polymer composites with excellent electromagnetic interference SE, which can be used in a wider range of applications.

In summary, the use of 3D printing technology to prepare electromagnetic interference shielding materials reduces the need for tedious processes such as traditional laboratory synthesis and provides materials with better smoothness. At the same time, it can promote the overlapping of fillers and the efficient construction of polymer composites in the network, which is an efficient and simple strategy for preparing polymer composites with high thermal conductivity and excellent EMI SE. In addition, because of its good electromagnetic shielding performance, 3D printing technology has great potential for use in the development of portable electronic devices. Most importantly, 3D printing technology can also be optimized by computer software, and can freely manufacture various structures, with adjustable performance and numerous structural design options. Therefore, it was expected to innovate different printing methods, resulting in increased porosity, more interfaces and higher strength, thereby enhancing the electromagnetic shielding performance. However, only a limited range of materials were suitable for 3D printers, and the influence caused by gaps between successive layers, viscosity problems, were also significant challenges, which may be the limiting factors impeding its use and its application expansion. It was believed that with the introduction of 4D and 5D printing in the manufacturing industry, the shortcomings of 3D printing technology could be eliminated and more advanced materials could be manufactured via these technologies. The use and improvement of 3D printing technology will soon bring about a new revolutionary world.

### 4.2. MXene-Based Membrance Shielding Materials

**Figure 15:**
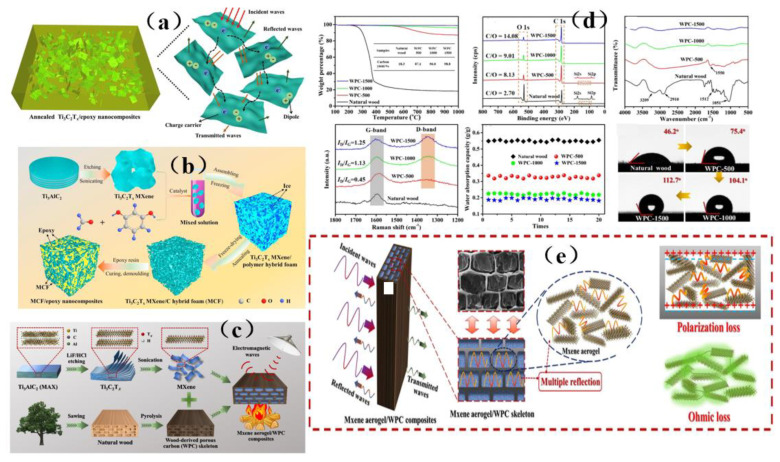
(**a**) Schematic diagram of shielding mechanism of annealed Ti_3_C_2_T_x_/epoxy resin electromagnetic interference shielding nanocomposite [130]. Copyright 2019, Elsevier. (**b**) Schematic diagram of preparation process leading to a MCF/epoxy electromagnetic shielding nanocomposite [131]. Copyright 2019, Elsevier. (**c**) Schematic diagram depicting the preparation depicting the MXene aerogel/WPC composite. (**d**) a: TGA, b: XPS, c: FTIR, d: Raman, e: water absorption capacity, f: water contact angles of natural wood WPC-500, WPC-1000 and WPC-1500. (**e**) a: Photos of “stucco brick” structural walls. b: SEM images of MXene aerogel /WPC composites, d: element mapping images of Ti, e: EMI SE of MXene aerogel /WPC composite, f: Comparison of EMI SE values between MXene aerogel /WPC composite and WPC-1500, g: electromagnetic interference and SE value are the same as density, h: Schematic diagram of electromagnetic wave passing through MXene aerogel /WPC composite. c, d, e [132]. Copyright 2020, Elsevier.

With the increasing popularity of portable and wearable devices, materials that are light in weight, have a low density, are highly flexible, and have excellent mechanical stability are becoming highly sought-after for use as EMI shielding materials. Due to its excellent conductivity, MXene has been widely investigated and applied in the field of electromagnetic interference shielding, and it has thus become a very popular electromagnetic interference shielding material [39,40,41,42]. However, during the application of MXene, it was reported that the EMI SE of MXene/polymer electromagnetic interference shielding composites was relatively low, randomly distributed MXene cannot readily form an effective conductive path in the polymer, resulting in poor shielding performance [130]. On the basis of ion intercalation and ultrasonic-assisted preparation of low-layer high-conductivity Ti_3_C_2_T_x_, Wang et al. [130] employed a low-temperature thermal reduction method to remove some polar groups from the surface of Ti_3_C_2_T_x_. A Ti_3_C_2_T_x_/epoxy electromagnetic interference shielding nanocomposite was subsequently prepared by a solution casting method, with shielding performance reaching as high as 41 dB. Research on its shielding mechanism shows that due to an impedance mismatch, part of the electromagnetic wave was reflected and absorbed by carrier interaction, and the rest was reflected and re-absorbed, thus causing more of the electromagnetic wave to be lost due to the presence of the shielding composite and fully attenuating the electromagnetic wave (Figure 15a). However, the demand for high shielding performance membrane shielding composites in the aerospace field was still facing challenges and further research was needed. In another study, Wang et al. [131] firstly prepared a low-level Ti_3_C_2_T_x_ MXene. Subsequently, porous Ti_3_C_2_T_x_ MXene/C composite foam (MCF) was prepared by a reduction method. An MCF/epoxy electromagnetic shielding nanocomposite with excellent shielding properties and mechanical properties was then prepared via vacuum-assisted impregnation and a curing process (Figure 15b). X-ray photoelectron spectroscopy (XPS) data show that MCF has a highly cross-linked network, so the membrane shielding material has robust mechanical properties. In addition, it has been found that absorption plays a dominant role in the shielding mechanism of MCF/ epoxy electromagnetic shielding nanocomposites, and the shielding performance reaches 46 dB (x band). MCF’s unique three-dimensional conductive network expands the range of applications for MXene membrane shielding materials into the field of electromagnetic interference shielding. Renewable porous biochar and two-dimensional MXene have attracted much attention in the field of high-end electromagnetic interference shielding because of their unique ordered structure and excellent conductivity. Liang et al. [132] prepared a MXene aerogel/wood-derived porous carbon (WPC) composite material with good conductivity and ultra-light weight properties by using porous carbon derived from natural wood (Figure 15c). Through a series of characterization methods (Figure 15d), it was demonstrated that the membrane is an excellent shielding material, and its shielding performance reaches as high as 71.3 dB. This kind of wall-like “mortar-brick” structure (in which the WPC skeleton is the “mortar” and the MXene aerogel is the “bricks”) not only effectively solves the structural instability of MXene aerogel network, but also greatly prolongs the propagation path of electromagnetic waves. It dissipates the incident electromagnetic waves in the form of heat energy and electric energy, thus showing superior EMI shielding performance (Figure 15e). This study is expected to provide a feasible way to prepare ultra-light, green, efficient and multifunctional MXene-based materials.

**Figure 16:**
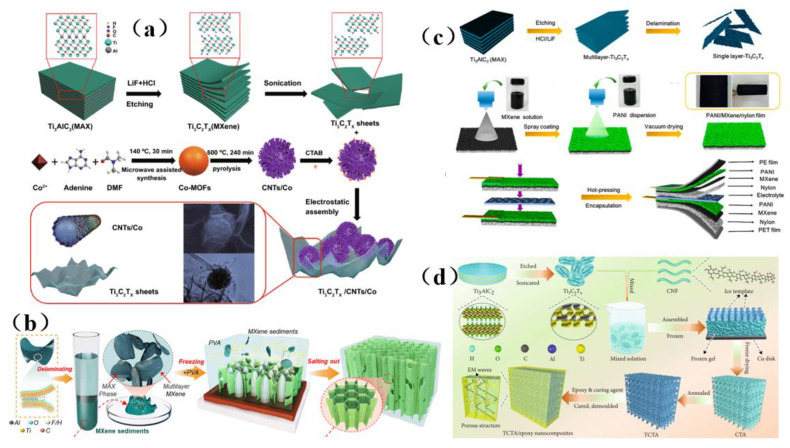
(**a**) Schematic diagram of the preparation pathway leading to a Ti_3_C_2_T_x_/CNTs/Co nanocomposite and performance evaluation of this material [133]. Copyright 2021, SpringerOpen. (**b**) Schematic diagram of preparation process of MS-based hydrogel [134]. Copyright 2022, American Chemical Society. (**c**) Schematic depiction of the preparation of multifunctional skin [135]. Copyright 2022, American Chemical Society. (**d**) Schematic diagram of TCTA/epoxy nanocomposites [136]. Copyright 2020, American Association for the Advancement of Science.

Although MXene membrane shielding materials have been widely used, how to use these materials as high-performance electromagnetic wave absorption and electromagnetic interference shielding materials and make them have multiple functions is still a great challenge. Zhen et al. [133] prepared a Ti_3_C_2_T_x_/carbon nanotubes/Co nanoparticle (Ti_3_C_2_T_x_/CNTs/Co) nanocomposite with a 2D/1D/0D structure via an electrostatic assembly method (Figure 16a). This membrane shielding material has highly integrated functions, including excellent electromagnetic wave absorption, EMI shielding efficiency, thermal cycle stability, photothermal conversion performance, flexibility, and hydrophobicity (Figure 16a). Similarly, hydrogels are rich in water pores, which are expected to promote the reflection of incident electromagnetic microwave (EMWs) and enhance the polarization loss ability of water molecules and hydrogen bond networks. This makes hydrogel promising as a high-performance EMI shielding material. Yang et al. [134] prepared a kind of electromagnetic interference (EMI) shielding material which integrates a honeycomb-like ordered porous structure, high conductivity MXene precipitates (MS)and water, and a highly flexible hydrogel, which is mainly composed of “garbage” MS and bionic pores (Figure 16b). This membrane shielding material not only had bionic ordered pore structure, but also possessed is a strong frame composed of highly crosslinked MS, which stabilizes the micron-sized pore structure and is more conducive to the formation of high-strength hydrogel with mechanical super-flexibility. Under the synergistic effect of this MS-based conductive network, 30 PVA chains, water and porous structure, MS-based hydrogel shows good EMI shielding performance, and more surprisingly, it has sensitive and reliable functions of human motion detection and intelligent coding. In addition, the pollution caused by the emission of thermal radiation also greatly affects human life. Therefore, there is an urgent need for multifunctional electronic skin with the functions of thermal radiation regulation and electromagnetic interference shielding. Song et al. [135] prepared a flexible electronics skin with Ti_3_C_2_T_x_ MXene as the conductive electrode (Figure 16c). It was found that this skin has flexible transmission power and high-performance shielding effectiveness of 36.3 dB. This technology could not have applicability in areas such as thermal radiation modulation and EMI shielding, but also provides technical and experimental guidance for the design of other multi-band spectral shielding materials. As part of their efforts to address the difficult processing and poor mechanical properties of MXene-based shielding materials, Wang et al. [136] prepared a three-dimensional highly conductive cellulose nanofiber/Ti_3_C_2_T_x_ MXene aerogel (CTA) with an oriented porous structure. They then thermally annealed the CTA to obtain thermally annealed (TCTA)/epoxy nanocomposites (Figure 16d). MXene and these materials were light weight, readily processable, moldable, had high EMI SE values, possessed excellent mechanical properties, and had good thermal stability.

In conclusion, the preparation or processing of these commonly used MXene layers for use in membrane shielding materials still faces several challenges such as high cost, low yield, or the need for additional functionalization, which limits the practical applicability of these materials due to the need for cost and environmental efficiency. Therefore, we should pay attention to the combination of composite membrane shielding materials, turn waste into treasure, and make it have the advantages of no waste, expansibility, and low cost. In addition, due to the weak interlayer interaction between MXene nanosheets, they tend to lack flexibility and poor mechanical strength. To address these issues, the best strategy is to compound MXenes with other components to achieve EMI shielding. Based on the above analysis, combined with multifunctional and excellent EMI shielding performance, MXene-based membrane shielding materials with high strength and superb flexibility have strong potential for applicability in the next generation of electronic products.

### 4.3. Carbon-Based Membrane Shielding Materials

Traditional shielding materials are prone to problems such as difficult processing, high cost, poor shielding effect, narrow absorption bands and secondary pollution. Carbon-based materials often have the advantages of electrical conductivity, easy molding, being light weight, having a low cost, providing corrosion resistance, as well as having wide absorption bands, and thus they have become the first choice of new generation shielding materials. Common examples of these carbon-based materials include graphite, graphene, carbon fibers, and carbon nanotubes [43,44,45,46]. Therefore, carbon-based shielding materials were often composite materials composed of carbon-based materials which serve as conductive fillers and other substrates [43,44,45,46]. The research on high-efficiency composite shielding materials by scholars all over the world has often focused on increasing the filling degree of carbon-based materials, improving the dispersion of fillers and reducing the thickness of composite materials [43,44,45,46].

**Figure 17:**
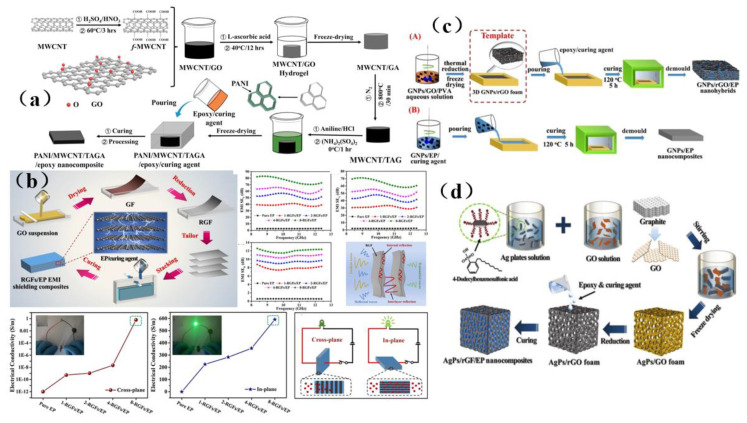
(**a**) Preparation schematic diagram and performance test of PANI/MWCNT/thermally annealed graphene aerogel/epoxy electromagnetic interference shielding nanocomposites [138]. Copyright 2019, Elsevier. (**b**) Schematic depiction of the fabrication process leading to RGF_S_/EP EMI shielding composite material [62]. Copyright 2019, Elsevier. (**c**) aA: Schematic diagram of the preparation pathway leading to three-dimensional GNPs/rGO/EP nanocomposites by the template method, bB: Schematic diagram showing the preparation of GNPs/EP nanocomposites by a traditional blending casting method [139]. Copyright 2019, Royal Society of Chemistry. (**d**) Schematic diagram showing the preparation of three-dimensional AgPs/rGF/EP nanocomposites [140]. Copyright 2019, Royal Society of Chemistry.

Graphene was a two-dimensional, single-layer material with a single-atom thickness. On account of its superior electrical properties and large aspect ratio, graphene has been highly valued as an electromagnetic interference shielding material. It was an allotrope of carbon with a sp^2^ configuration and has excellent mechanical and thermal properties. Graphene has been widely used as an electromagnetic interference shielding material in ultra-thin flexible membrane, paper, laminates, microcellular foams, sheets, and other materials [137]. The electromagnetic shielding efficiency of graphene can reach as high as 135 dB, while the value required for commercial application was only 20 dB [137]. Huangfu et al. [138] prepared a membrane shielding nanocomposite containing graphene oxide (Figure 17a). The prepared nanocomposite had a porous structure, which provided the shielding material with good conductivity (52.1 S/m), shielding efficiency (42 dB), mechanical properties (5.35 GPa) and thermal properties (171.3 °C). Unfortunately, carbon-based fillers do not readily form an effective continuous network, which makes it difficult for electrons to transport in an epoxy resin matrix and impedes efforts to improve the EMI SE values. However, the preparation of a continuous structure by a pre-forming process can significantly improve EMI SE value. Liang et al. [62] successfully prepared graphene oxide/epoxy resin (RGF/EP) electromagnetic shielding composites by introducing different layers of graphene oxide membranes into an epoxy resin matrix by pre-arrangement. It was found that the introduction of highly aligned RGF with a layered structure helps to improve the EMI SE and in-plane conductivity of RGF/EP electromagnetic shielding composites (Figure 17b). Designing a predictable microstructure and significantly improving its shielding effectiveness against electromagnetic interference is still a daunting challenge. Liang et al. [139] prepared a three-dimensional porous graphene nanosheet/reduced graphene oxide foam/epoxy resin (GNPs/reduced graphene oxide/EP) nanocomposite (Figure 17c). The shielding performance of this material reached as high as 51 dB, the thermal conductivity was 1.56 W/mK, and the electrical conductivity was as high as 179.2 S/m. The study provides a new design strategy for shielding and efficient heat dissipation of multifunctional carbon-based composites. Liang et al. [140] also designed a three-dimensional silver sheet/reduced graphene oxide foam (AgPs/RGF) with many regular spherical hollow structures, and successfully prepared a three-dimensional AgPs/RGF/EP nanocomposite with a highly regular separation structure (Figure 17d), which can shield 99.998% of electromagnetic waves and has a minimal skin depth. The design of this material demonstrates a promising way to prepare lightweight and high-precision electronics for key applications such as those in the aerospace sector.

**Figure 18:**
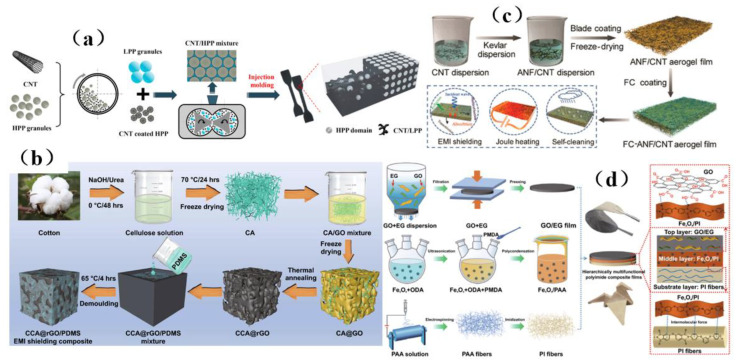
(**a**) Schematic diagram showing the preparation of separated carbon nanotubes/polypropylene composites and their performance evaluations [141]. Copyright 2020, Elsevier. (**b**) Schematic diagram of the manufacturing process leading to CCA@RGO/PDMS electromagnetic interference shielding composite material [142]. Copyright 2021, SpringerOpen. (**c**) Schematic diagrams showing the preparation, and multifunctional characteristics of FC-ANF/CNT mixed gas gel membrane [45]. Copyright 2019, Elsevier. (**d**) Schematic diagram of preparation process of PI composite membrane [143]. Copyright 2020, American Chemical Society.

The separation structure formed in carbon-based composites has great advantages in improving the electromagnetic interference shielding performance. However, due to the limitation of processing methods and the severe deterioration of mechanical properties, t the practical applicability of this composite is limited. Zhang et al. [141] prepared a separated carbon nanotube/polypropylene (CNT/PP) composite by simple and environmentally friendly methods such as pre-coating, melt mixing, and injection molding (Figure 18a). The material not only has good at shielding performance, but also has good tensile strength and a desirable Young’s modulus, which provides a feasible method for the separation and compounding of carbon-based shielding materials. Song et al. [142] prepared a CCA@rGO/PDMS EMI shielding composite materials using a backfilling method (Figure 18b), and obtained the best shielding performance of 51.0 dB, excellent thermal conductivity, robust mechanical properties, and good thermal conductivity. This excellent comprehensive performance suggests that cellulose carbon aerogel (CCA)@rGO/PDMS electromagnetic shielding composite material may have prospects for use in light and flexible electromagnetic shielding composite materials. In addition, thin membranes are an ideal choice for electromagnetic interference shielding because of their ultra-thin planar structure, light weight properties, good flexibility, and facile preparation process. Hu et al. [45] prepared a multifunctional aerogel membrane composed of strong aramid nanofibers (ANFs), conductive carbon nanotubes (CNTs) and hydrophobic fluorocarbon (FC) resin (Figure 18c), which has a large specific surface area (232.8 m^2^ G1), high conductivity (230 S·m^−1^), and excellent hydrophobicity (its contact angle can reach 137°). Guo et al. [143] prepared a multi-functional layered carbon-based composite membrane with graphene oxide/expanded graphite (GO/EG) as the top heat conduction and EMI shielding layer by adopting the layered design and assembly strategy (Figure 18d). The composite membrane has a high in-plane thermal conductivity (95.40 W (m·K)^−1^), an excellent EMI shielding effect (34.0 dB), good tensile strength (93.6 MPa) and fast electric heating response (5 s), and is thus a promising candidate for a broad range of applications

As the lightest and thinnest material known in the world, carbon-based materials have excellent conductivity, high aspect ratios, large specific surface areas, abundant functional groups, ultra-light weight, and thus they have great application potential as electromagnetic/radiation shielding materials. Carbon-based membrane shielding materials have high thermal conductivity, high inherent tensile strength, and high elastic moduli, which provides them with unique advantages for applications as multifunctional electromagnetic shielding materials. In order to ensure that these types of membrane shielding materials can better meet future technological needs, there are still many challenges that must be addressed, such as reducing their cost, and improving their flexibility, corrosion resistance, thermal conductivity, transparency, and environmental stability. In the future, researchers will still need to develop new methods, find new materials, and optimize the construction technology of multiple composite structures to effectively improve the performance of carbon-based electromagnetic shielding materials, and make them multifunctional (high thermal conductivity, corrosion resistance, and high transparency) and intelligent on the basis of meeting the performance requirements of “thinness, lightness, strength, and width”. Successfully achieving these goals will greatly expand the applicability of carbon-based electromagnetic shielding materials. A wide range of carbon-based materials were highly favored for their excellent properties, such as being light weight, having excellent flexibility, extraordinary electrical properties and corrosion resistance, which make great contributions to electromagnetic interference shielding and other applications. The disadvantage of carbon-based electromagnetic interference shielding materials is their low impedance matching caused by high conductivity, which leads to high reflection and low absorption.

### 4.4. Iron-Based Membrane Shielding Materials

Due to impedance mismatch, most electromagnetic waves will be reflected at the interface between the composite and the air, which will cause electromagnetic pollution to to be released into the surrounding environment [144,145]. The research shows that the introduction of magnetic materials can improve the impedance matching performance between composite materials and air, weaken the reflection of electromagnetic waves, and absorb electromagnetic waves through magnetic loss [146,147]. Depositing magnetic nanoparticles (Fe_2_O_3_, Fe_3_O_4_, NiFe_2_O_4_, etc.) onto conductive fillers can not only enhance their EMI SE, but also achieve insulation modification and solve the contradiction between excellent electromagnetic interference shielding and electrical insulation performance [148].

**Figure 19:**
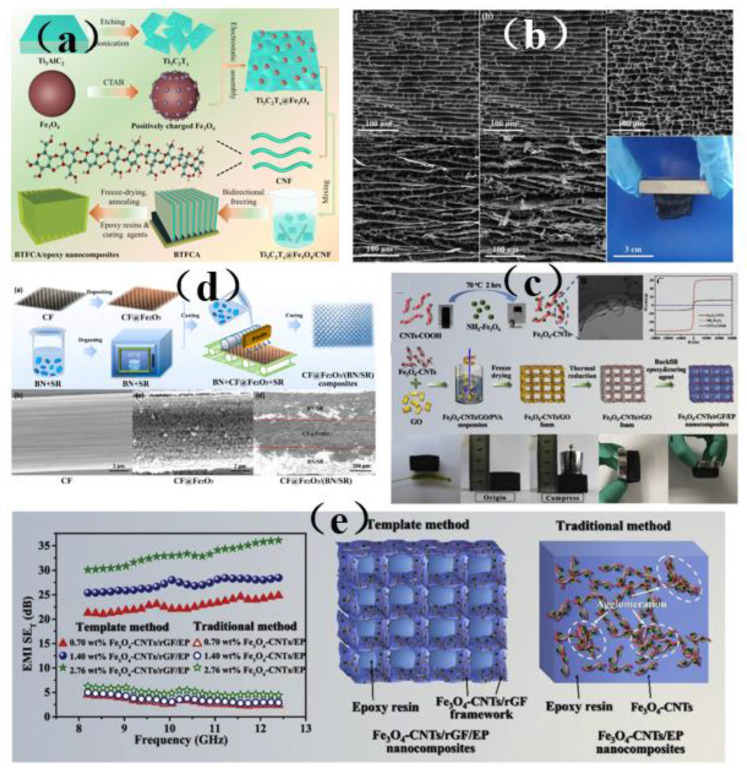
(**a**) A schematic diagram of the preparation process leading to the BTFCA/epoxy nanocomposite. (**b**) Morphology of BTFCA and BTFCA/ epoxy nanocomposites. a, b [149]. Copyright 2022, SpringerOpen. (**c**) Schematic diagram and microstructure of CF@Fe_2_O_3_/(BN/Sr) composites [148]. Copyright 2022, Elsevier. (**d**) Operating temperature of CPU. (**e**) Comparison of EMI SET of Fe_3_O_4_—CNTs/EP and 3D Fe_3_O_4_—CNTs/rGF/EP nanocomposites physically blended by different preparation methods. d, e [85]. Copyright 2019, Elsevier.

Wang et al. [149] used a Ti_3_C_2_T_x_@Fe_3_O_4_/CNF aerogel (BTFCA) to prepare BTFCA/epoxy nanocomposites with a long-distance layered structure (Figure 19a). Due to the introduction of Fe_3_O_4_, BTFCA is endowed with excellent magnetism. In addition, the composite can retain its original long-distance layered structure and maintain structural integrity. This is mainly because the high rigidity of Fe_3_O_4_ also provides BTFCA with a high degree of rigidity, which allows BTFCA/epoxy nanocomposites to retain the integrity of the layered structure arranged remotely (Figure 19b). In order to improve the electrical insulation performance of electromagnetic interference, the influence of internal conductive materials can be reduced by the structural design of composite materials and the insulation of the outer layer of the sandwich structure. Guo et al. [148] prepared a sandwich structure of CF@Fe_2_O_3_/(BN/Sr) composite material by depositing Fe_2_O_3_ particles onto carbon fiber (CF) with CF @Fe_2_O_3_ as filler (Figure 19c). Its structure can not only realize the heat conduction and electrical insulation functions of composite materials, but also achieve excellent EMI shielding performance and reduce secondary electromagnetic pollution through an “absorption-reflection (transmission)-reabsorption” process when electromagnetic waves pass through conductive fillers bearing magnetic materials. In addition, the CF@Fe_2_O_3_/(BN/SR) composite material was found to exhibit a better heat dissipation effect (5.6 °C) than commercial silicone grease (QM850) when it was evaluated on a computer CPU as a test platform. In addition, it has wide-ranging application prospects in the electronics field. As dipolar materials, Fe_2_O_3_ materials polarize in the presence of these waves, which leads to higher attenuation of electromagnetic waves. In order to efficiently construct a three-dimensional magnetic graphene-based composite structure and significantly improve electromagnetic interference, Liang et al. [85] prepared a three-dimensional Fe_3_O_4_-modified carbon nanotube/reduced graphene oxide foam/epoxy (3 Fe_3_O_4_-CNTs/RGF/EP) nanocomposite (Figure 19d). Subsequent performance tests revealed that its conductivity reaches 15.3 S/m and its EMI SE value reaches 36 dB, which is increased by nearly 482% compared with the composite material, which lacks a 3D structure. The introduction of Fe_3_O_4_ nanoparticles in the study will increase the magnetic and dielectric losses, on the one hand, due to the interface polarization between Fe_3_O_4_ nanoparticles, and other materials, and on the other hand, due to the formation of heterogeneous systems and stronger coupling between adjacent Fe_3_O_4_ nanoparticles, which will polarize in the presence of electromagnetic fields, thus obtaining better electromagnetic wave absorption. Therefore, excellent magnetism and efficient three-dimensional skeleton structure were the primary factors for the excellent electromagnetic shielding performance of three-dimensional Fe_3_O_4_-CNTs/rGF/EP nanocomposites (Figure 19e).

**Figure 20:**
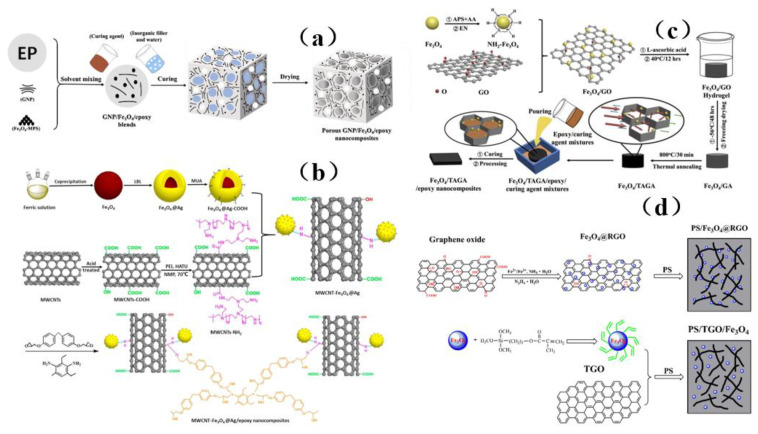
(**a**) Schematic diagram of porous GNP/ Fe_3_O_4_/epoxy nanocomposites prepared by epoxy-water-inorganic filler suspension emulsion polymerization [43]. Copyright 2019, Elsevier. (**b**) Schematic diagram of general preparation process of MWCNT-Fe_3_O_4_@Ag/epoxy resin nanocomposite [150]. Copyright 2019, Elsevier. (**c**) Preparation schematic diagram of Fe_3_O_4_/TAGA/epoxy nanocomposite [151]. Copyright 2018, Elsevier. (**d**) Schematic diagram of the synthesis of (a) PS/ Fe_3_O_4_@RGO and (**b**) PS/TGO/Fe_3_O_4_ composites [3]. Copyright 2015, Elsevier.

Although iron oxide has excellent magnetic properties and strong spin polarization at room temperature, it can be used to absorb microwave radiation. However, Fe_3_O_4_ nanoparticles tend to aggregate due to their strong magnetic dipole-dipole interaction, which affects the shielding effectiveness. To solve these problems, Liu et al. [43] prepared a three-dimensional porous graphene/Fe_3_O_4_/epoxy nanocomposite (Figure 20a), which can effectively prevent agglomeration and has excellent thermal stability as well as mechanical properties. Wang et al. [150] functionalized Fe_3_O_4_ nanoparticles with silver and 11 mercaptononanoic acid (MUA), and they reacted Fe_3_O_4_@Ag-COOH nanoparticles with the acyl amine of MWCNTs-NH_2_ to obtain conductive and magnetic layered composite nanoparticles MWCNT-Fe_3_O_4_@Ag (Figure 20b). The functionalized nanoparticles were readily dispersible in the composite material. Yiming et al. [151] used ethylenediamine functionalized Fe_3_O_4_ (NH_2_-Fe_3_O_4_) nanoparticles and graphene oxide (GO) to prepare a composite material with high shielding effectiveness (Figure 20c), which can be readily dispersed with graphene to obtain excellent shielding performance. Chen et al. [3] prepared PS composites with high electrical conductivity and electromagnetic shielding effectiveness by blending modified Fe_3_O_4_ nanoparticles with other solutions (Figure 20d). The modified nanoparticles were easily dispersed in the solution, exhibiting a synergistic effect with other materials, showing a good microwave absorption effect, and greatly enhancing the shielding performance.

**Figure 21:**
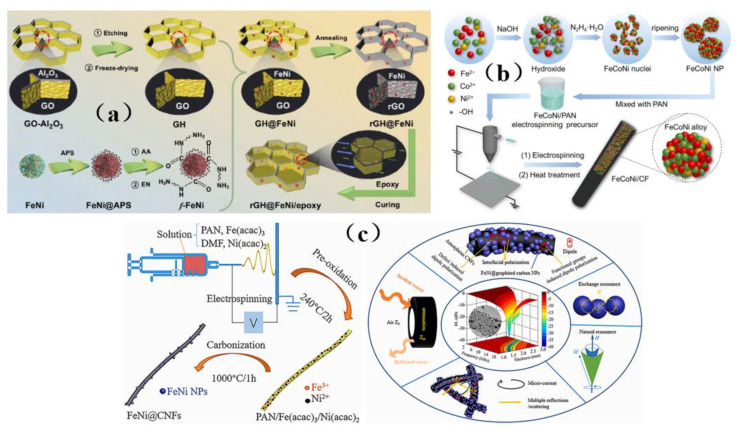
(**a**) Schematic diagram showing the preparation of an RGH@FeNi/epoxy composite [152]. Copyright 2022, SpringerOpen. (**b**) Schematic diagram of the synthesis strategy leading toFeCoNi/CF composites [153]. Copyright 2022, SpringerOpen. (**c**) Schematic diagram and mechanism diagram of the manufacturing process leading to FeNi@CNFs [154]. Copyright 2022, Elsevier.

Besides iron oxide, the FeNi alloy also has excellent initial permeability, relative permeability, low coercivity, as well as repeated magnetization loss, and thus it has great potential in EMI shielding applications. Song et al. [152] loaded functionalized FeNi alloy particles (f-FeNi) on a graphene oxide aerogel with a regular honeycomb structure (GH), and they prepared a magnetic and conductive rGH@FeNi/epoxy electromagnetic shielding composite (Figure 21a). Its shielding efficiency is as high as 46 dB, and at the same time, it has good thermal stability (its heat-resistance index and temperature at the maximum decomposition rate were 179.1 °C and 389 °C, respectively. Yang et al. [153] embedded a FeCoNi medium entropy alloy in a one-dimensional carbon matrix frame to prepare a composite electromagnetic wave absorber (Figure 21b), which significantly enhanced the electromagnetic wave absorption performance. Guan et al. [154] prepared highly dispersed fine FeNi nanoparticles (NPs) that were coated with carbon nanofibers (FeNi@CNFs) (Figure 21c), for use in shielding materials to obtain a satisfactory synergy of impedance matching and attenuation resistance. The use of FeNi NPs was an effective and promising strategy for designing light and high-performance electromagnetic wave absorbers.

In short, from the perspective of application, the membrane shielding materials incorporating iron and its oxides are very useful in energy, medical treatment, research, and many other fields. In this section, we have explored the composite materials composed of carbon, polymer, and iron-based materials, in which iron is an important component that can prevent electromagnetic interference (EMI) through reflection and absorption. Dielectric loss and magnetic loss are the reasons for high microwave absorption and total shielding performance. In this case, iron and its components can be combined with conductive polymers, carbon-based materials, or other materials to achieve a synergistic effect, which has become a popular strategy for EMI shielding applications. This approach also likely points the way for the future development of electromagnetic shielding materials that are lighter, thinner, less costly, and offer superior absorption performance compared to that of the existing materials.

### 4.5. Cellulose-Based Membrane Shielding Materials

At present, most membrane shielding materials are made by combining highly conductive elements with substrates by various methods, such as coating or mixing. However, numerous reported membranes shielding materials were based on non-renewable polymers [39,40,41,42,43,44,45,46,47,48,49,50], which is inconsistent with the concept of green chemistry and sustainable development. Therefore, some biopolymers have attracted the attention of researchers [54,155]. As the most abundant renewable polymer on earth, cellulose is widely used in various fields because of its excellent characteristics, including low production cost, biodegradability, biocompatibility, and being light in weight [156]. Cellulose contains many hydroxyl groups, which may promote the formation of hydrogen bonds, which can promote the combination of cellulose with other elements. Generally, cellulose composites are prepared in the form of membranes, papers, or porous materials. Cellulose serves as a matrix in composites, which can improve the mechanical properties of these materials. At the same time, it can be used as a dispersant to evenly distribute nanoparticles and reduce the content of conductive components, thus yielding a thin and efficient EMI shielding material [155].

**Figure 22:**
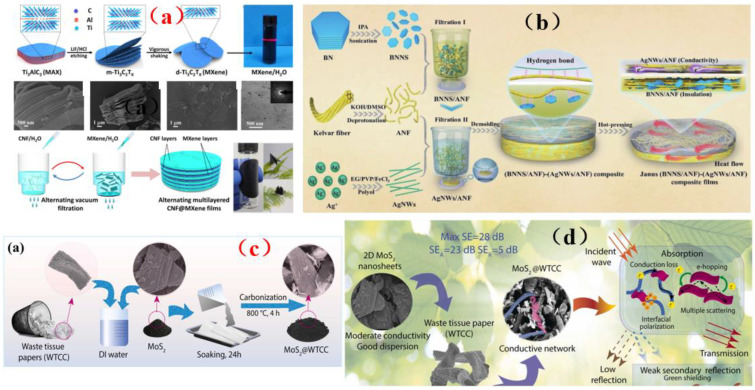
(**a**) A schematic diagram of the preparation of alternating multilayer membrane (CNF@MXene) [70]. Copyright 2020, American Chemical Society. (**b**) A schematic diagram of the preparation of (BNNS/ANF) -(AgNWs/ANF) thermal conductive composite membrane with Janus structure [157]. Copyright 2022, Tsinghua University Press. (**c**) A schematic diagram depicting the synthesis of cellulose carbon (MoS_2_@WTCC) derived from waste paper towels decorated with MoS_2_. (**d**) The low conductivity of MoS_2_ and cellulose fiber structure produce an effective conductive network, which is essential to improve shielding performance and minimize secondary reflections. c, d [158]. Copyright 2022, Elsevier.

Zhou et al. [70] designed a multilayer membrane with an alternating structure comprised of cellulose nanofiber (CNF) layers and MXene layers by alternating vacuum filtration (Figure 22a). Based on the mechanical frame effect of the CNF layers, the nano-zigzag cracks in the MXene layer can be effectively prevented from spreading to the whole membrane, and the mechanical strength and toughness of the alternating multilayer membrane (CNF@MXene) are improved. It can withstand more than 1000 folding tests without becoming damaged, and the shielding efficiency was as high as 40 dB, paving the way for the application of new intelligent protection equipment suitable for cold and complex conditions. Han et al. [157] compounded aramid nanofibers (ANFs) that were prepared by a chemical dissociation method with other fillers, thus obtaining a thermally conductive and electromagnetic interference shielding composite membrane (Figure 22b) with a Janus structure (boron nitride nanosheets (BNNS)/ANF). This BNNS/ANF film exhibits both conducting as well as insulating behavior and has excellent electrical stability and reliability. Uddin et al. [158] simply soaked, carbonized, and integrated two-dimensional layered MoS_2_ with low conductivity into waste cellulose paper, which promoted absorption by optimizing the dielectric loss and green shielding by reducing reflection. This was mainly due to the introduction of two-dimensional stacked MoS_2_ sheets, which provided the source of interfacial polarization and multiple relaxation pathways. MoS_2_ also acts as a bridge between cellulose fibers, forming a conductive network, promoting conductive loss, and thus providing an effective strategy for sustainable manufacturing of high-performance green EMI shielding materials (Figure 22c,d).

**Figure 23:**
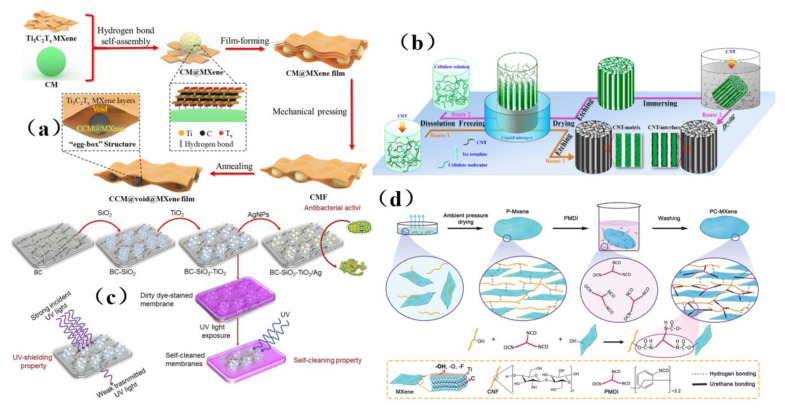
(**a**) Schematic diagram showing the fabrication of CCM@void@MXene composite membrane (CVMF) [42]. Copyright 2021, Elsevier. (**b**) Schematic depiction of the preparation process leading to carbon nanotube matrix/cellulose porous composite (route 1) and carbon nanotube interface/cellulose porous composite (route 2) [159]. Copyright 2019, American Chemical Society. (**c**) Schematic diagram showing the preparation of cellulose-based membrane shielding material [160]. Copyright 2020, Elsevier. (**d**) Schematic diagram of PC-MXene physical and chemical double cross-linking preparation process [161]. Copyright 2021, WILEY-VCH Verlag GmbH & Co. KGaA, Weinheim.

Many studies show that the internal multilayer structure provides an important contribution toward shielding effectiveness. Qian et al. [42] designed a structure in which carbonized cellulose microspheres were inserted into the Ti_3_C_2_T_x_ MXene layer (CCM@MXene). A CCM@void@MXene composite membrane with an “egg carton” structure was obtained (Figure 23a). Cellulose can be integrated with Ti_3_C_2_T_x_ MXene to form a graded material to enhance microwave absorption or EMI shielding performance, and then show better conductivity and shielding efficiency. This graded porous egg box-shaped structure was a promising candidate for use in high-efficiency EMI shielding systems. Zhang et al. [159] designed a CNT interface/cellulose porous composite (Figure 23b) by adjusting the porous microstructure and the distribution of carbon nanotubes in the cellulose composite and achieved excellent shielding performance as well as good mechanical properties and low density. Its shielding effect reached 40 dB, and its modulus was 279 MPa g^−1^ cm^3^. This research can preserve the environment and pave an effective way for high-performance electromagnetic interference shielding materials, thus promoting many practical and advanced applications of cellulose. Rahman et al. [160] designed a cellulose-based membrane shielding material based on bacterial cellulose (BC), a flexible and multifunctional organic-inorganic hybrid membrane (BC-SiO_2_-TiO_2_/Ag) (Figure 23c). This material can be easily disinfected under ultraviolet irradiation from a lamp or natural light, and safely discarded or even recycled. Wu et al. [161] used superfine (1.4 nm) cellulose nanofibers to achieve physical and chemical cross-linking of MXene (PC-MXene) nanosheets, thus preparing PC-MXene membranes with good flexibility and high conductivity (Figure 23d). The addition of nano-cellulose reduces the insulation polymer gap between MXene nano-sheets, thus preventing the deterioration of the conductivity and EMI shielding performance of MXene/ polymer composites and enabling the fabrication of strong and ultra-thin film shielding materials.

As electromagnetic shielding and absorbing materials, cellulose-based composites provide impressive performance while also having room for further expansion in terms of their performance, applications, and structural diversity. Besides their desirable characteristics, such as their low density, low cost, and good electromagnetic efficiency, other requirements such as corrosion resistance, thermal stability, and hydrophobicity cannot be ignored due to the diverse range of working environments that should also be considered. Therefore, there is still much room for innovation and growth in the research of cellulose-based materials in many aspects, and the road to maturity will be difficult and full of expectations. A helpful area for further research will be to gain deeper insight into the shielding mechanism of cellulose-based composites, and this knowledge will provide more new opportunities leading to the next generation of electromagnetic absorption shielding materials.

### 4.6. New Lead-Free Membrane Shielding Materials

Over the years, researchers at home and abroad have conducted a series of investigations on radiation shielding materials, and various radiation shielding materials have been developed. However, further research was needed to optimize the preparation process, enhance the radiation shielding performance, and improve the comprehensive performance of these materials [162,163]. Traditional radiation protection materials used for personal radiation protection have been rubber-based composite materials with lead and its compounds as the main filling materials. Because of their poor softness, strong toxicity, limited shielding effect, and other problems (especially their heavy weight and poor comfort), these materials cannot meet the actual requirements for safe and comfortable protective clothing [162,163]. In order to obtain ideal protective materials, it is necessary to prepare lead-free radiation protection composites with better flexibility and radiation shielding performance by optimizing composite component design and improving composite processing technology.

**Figure 24:**
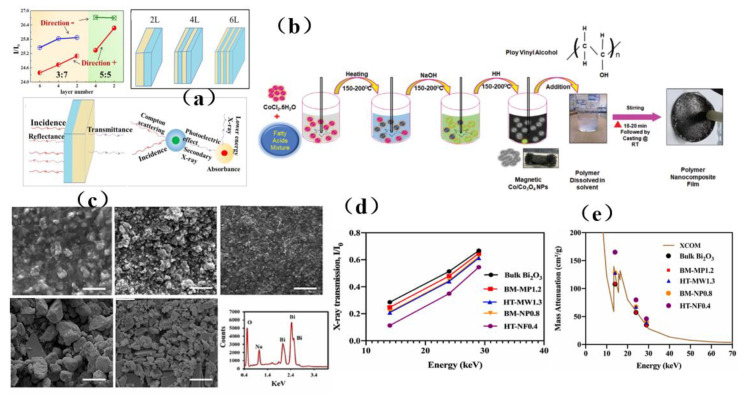
(**a**) a: Relationship between X-ray transmittance and layer thickness ratio (4:6 and 3:7), incident direction and number of layers, b: Structural schematic diagram of a multilayer composite materials with different layers, c: Schematic diagram of a photon attenuation mechanism [164]. Copyright 2021, Springer Nature. (**b**) Synthesize NPs, and then make thin membrane [165]. Copyright 2020, American Scientific Publishers. (**c**) SEM image of the top section of the prepared thin membrane and EDX analysis of Bi_2_O_3_ bulk [166]. Copyright 2021, Elsevier. (**d**) X-ray transmission of bulk Bi_2_O_3_, BM—MP1.2, HT—MW1.3, BM—NP0.8 and HT—NF0.4 [166]. Copyright 2021, Elsevier. (**e**) Get the mass attenuation coefficient of bulk Bi_2_O_3_ from XCOM, and comparison with the corresponding value of each experimental sample. c, d, e [166]. Copyright 2021, Elsevier.

In order to solve the lead pollution caused by the widespread use of lead-containing materials in shielding materials for radiation protection, technical approaches for utilizing lead-free composite shielding materials have been put forward. Li et al. [164] designed a lead-free multilayer polymer composite, which is a layered composite based on tungsten/octene copolymer)/(bismuth/octene copolymer). The shielding mechanism of this structure was primarily due to X-ray penetration becoming weakened by the synergistic effect of layers and interfaces, so that the X-ray shielding ability can be effectively enhanced (Figure 24a). Tiwari et al. [165] prepared a nano-composite membrane with a green surfactant (Figure 24b), which has excellent shielding effectiveness and is very suitable for commercial applications. Yu et al. [166] studied the influence of micro-nano Bi_2_O_3_ membranes with different morphologies on shielding performance, and they found a synergistic effect between the particle size and the morphology on low-energy X-ray attenuation (Figure 24c–e). Therefore, the synergistic effect of particle size and morphology should be considered during the design of effective radiation-proof clothing.

In summary, the current research mainly focuses on the lead-free radiation shielding materials that can be used as radiation shielding materials. According to the cost performance, practicability and physical properties of lead-free materials, there are many kinds of lead-free materials that can be suitable for applications as radiation shielding materials. Lead-free radiation-proof materials are lightweight and have good protective performance. Therefore, future medical radiation protection clothing must be light, efficient and environmentally friendly, so as to improve the working comfort for medical staff, ensure their health and safety, and not contaminate the environment with lead or other toxic materials. Finally, no matter what kind of shielding agent is added to medical radiation protective clothing, in addition to its radiation protection performance, many factors such as mechanical properties, preparation difficulty, impact on the environment, cost, and so forth. should be considered to meet the national standards of medical protective clothing. 

## 5. Mechanism of Membrane Shielding Materials

**Figure 25:**
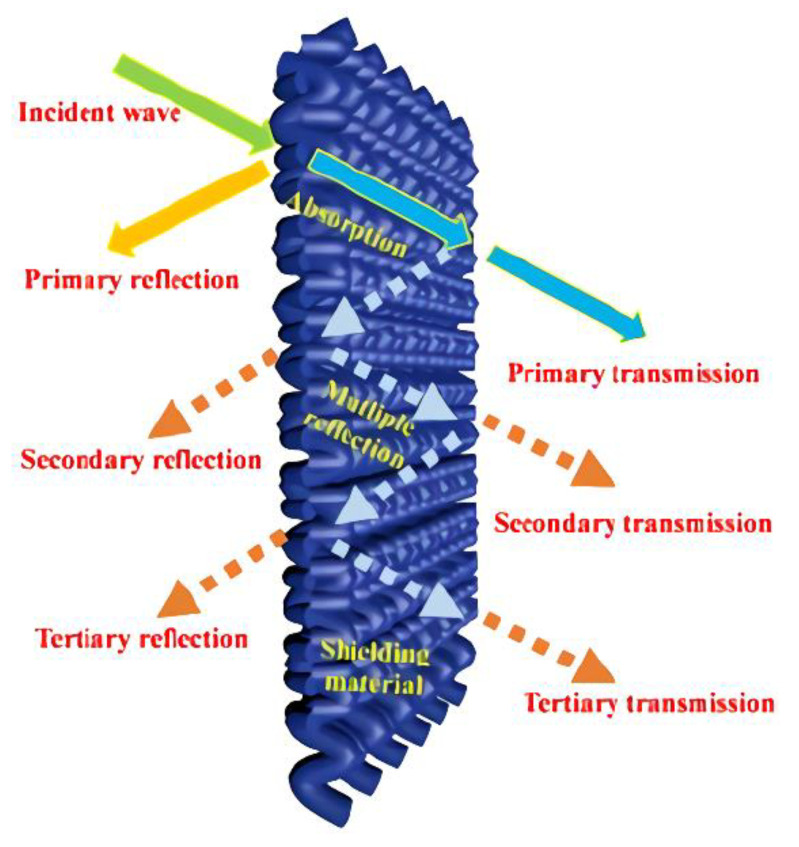
Possible interaction between electromagnetic/radiation and shielding materials [168].

In view of the increasingly serious electromagnetic/radiation pollution, the research, development, and application of membrane shielding materials have garnered significant attention in various fields. Electromagnetic/radiation shielding materials refer to materials that attenuate electromagnetic waves through reflection, multiple reflection, and absorption, and cut off or reduce the transmission of electromagnetic/radiation waves [54]. Their mechanism of action was different from that of wave absorbing materials [54]. As can be seen from Figure 25, when the incident electromagnetic wave reaches the surface of the shielding material from the emission source, due to the impedance change of the propagation medium, part of the electromagnetic wave is reflected back to the space on the same side of the emission source, and the attenuation of the electromagnetic wave caused by this is called reflection loss. The reflection loss value was proportional to the interface impedance difference. Subsequently, the remaining electromagnetic waves within the shielding body were further absorbed by the shielding material through dielectric loss or magnetic loss (absorption loss) or attenuated through multiple reflections (multiple reflection loss), and finally a small amount of electromagnetic waves passed through the shielding material to reach the reflection source [167]. The ability of a material to reflect, absorb, and attenuate radiation was closely related to its own electronic and magnetic properties. For conductive shielding materials, increasing conductivity can enhance absorption and reflection loss at the same time [63]. Generally, the shielding effectiveness or electromagnetic wave attenuation rate can be used to evaluate the shielding performance of materials. Shielding efficiency was the result of the joint action of three attenuation modes, and this value is expressed in decibel (dB) units. The larger the shielding value, the better the electromagnetic wave blocking effect. However, the electromagnetic waves reflected back to the same side of the emitting source or transmitted through the shielding material will continue to endanger human health, interfere with the operation of equipment, and cause secondary pollution. At present, a growing amount of research is devoted to reducing the proportion of reflection loss, improving the absorption efficiency of shielding materials, and reducing the transmission coefficient of shielding materials.

**Figure 26:**
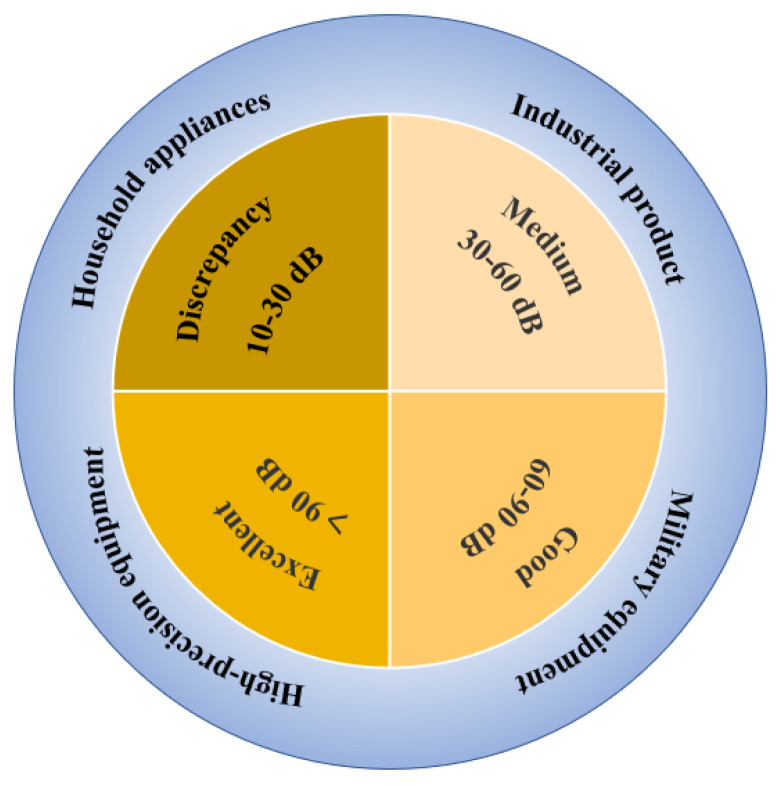
Classification of shielding levels.

Shielding efficiency was the most intuitive index to measure the performance of shielding materials [54]. If a material’s shielding efficiency is less than 10 dB, it can be considered to have no shielding efficiency. If the shielding efficiency was below 30 dB, the material was considered to have poor shielding performance. A material with a shielding efficiency in the range of 30–60 dB was considered to have moderate performance which can meet the requirements of civil, general commercial, or industrial electronic equipment. At 60–90 dB, the shielding material was considered to have high shielding performance and could be used for military and aerospace applications. When a material’s shielding efficiency was greater than 90 dB, it was considered to have excellent shielding performance, and was suitable for demanding scenarios such as the shielding of high-precision equipment [63]. According to their shielding effectiveness, shielding materials can be divided into different categories of shielding levels, as shown in Figure 26.

According to the mechanism of electromagnetic shielding materials, the total electromagnetic shielding effectiveness (*SE_Total_*) can be divided into three parts as expressed in Equation (1) [54,63,169,170]. The first of these parts is a reflection loss (*SE_R_*) and is shown in Equation (2), which refers to the loss caused by the impedance mismatch of electromagnetic waves on the surface of electromagnetic shielding materials. The second part is the absorption loss (*SE_A_*) as shown in Equation (3), which refers to the loss caused by the absorption of the energy of electromagnetic waves by electromagnetic shielding materials during transmission within these materials after the electromagnetic waves enter the materials. *SE_A_* is further sub-divided into electrical loss $\tan\delta_{\mu }$ which is expressed by Equation (4) and magnetic loss $\tan\delta_{\varepsilon }$ which is expressed by Equation (5). The third category is multiple reflection loss (*SE_MR_*), which is expressed by (6). This loss is caused by multiple reflections between the inner walls of electromagnetic shielding materials, and it should be noted that *SE_MR_* can be omitted when the electromagnetic shielding effectiveness is greater than 15 dB.

(1) $$SE_{Total}=SE_{R}+SE_{A}+SE_{MR}$$

(2) $$\begin{array}{ll} SE_{R}=20\log\frac{\eta_{0}}{\eta_{s}} & =39.5+10\log\sqrt{\frac{\sigma }{2\pi f\mu }}=50+10\log\frac{\sigma }{f}\\ & =10\log\frac{1}{1-R} \end{array}$$

(3) $$SE_{A}=20\log e^{\frac{d}{\delta }}=8.7\log\sqrt{\pi f\mu \sigma }=10\log\frac{1}{1-R}$$

(4) $$\tan\delta_{\mu }=\frac{\mu^{\prime \prime }}{\mu^{\prime }}$$

(5) $$\tan\delta_{\varepsilon }=\frac{\varepsilon^{\prime \prime }}{\varepsilon^{\prime }}$$

(6) $$SE_{MR}=20\log\left(1-e^{\frac{2d}{\delta }}\right)$$

In the above equations, *η*_0_ and *η_s_* represent the inherent impedance of the propagation medium and material, respectively. Meanwhile, *ε′* (the real part of the dielectric constant) and *μ′* (the real part of permeability), respectively, represent the material’s ability to store electromagnetic waves, while $\varepsilon^{\prime \prime }\;$(the imaginary part of dielectric constant) and $\mu^{\prime \prime }$ (the imaginary part of permeability) represent the material’s ability to lose electromagnetic waves. In addition, *δ* denotes the skin depth, *d* is the material thickness, *σ* represents the electrical conductivity, *f* denotes the electromagnetic wave frequency, *μ* is the magnetic permeability, while *R* and *T* denote the reflection coefficient and transmission coefficient, respectively. 

**Figure 27:**
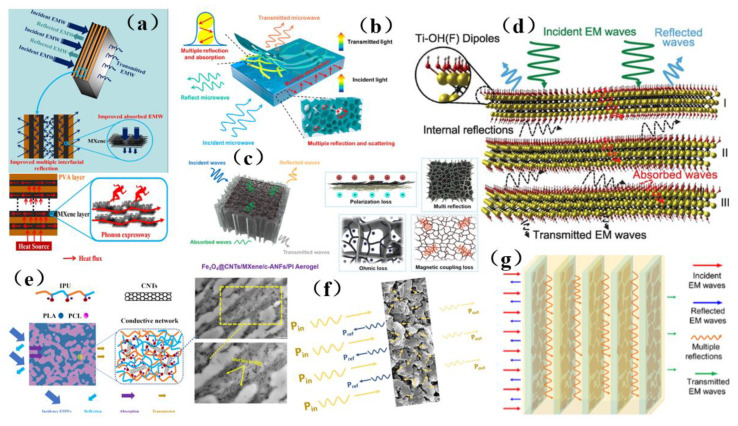
(**a**) Electromagnetic microwave dissipation mechanism and heat conduction mechanism with high thermal conductivity [170]. Copyright 2020, Elsevier. (**b**) Illustration of the launching mechanism [171]. Copyright 2022, American Chemical Society. (**c**) Electromagnetic interference shielding mechanism of composite aerogel [172]. Copyright 2022, American Chemical Society. (**d**) Electromagnetic interference shielding mechanism [173]. Copyright 2016, American Association for the Advancement of Science. (**e**) Schematic diagram of an electromagnetic interference shielding mechanism [174]. Copyright 2022, Ivyspring International Publisher. (**f**) Schematic diagram of the transformation of co-continuous conductive structure and its influence on the interaction with incident waves [175]. Copyright 2020, Elsevier. (**g**) Schematic diagram of the EMI shielding mechanism of conductive pearls [177]. Copyright 2022, Wiley.

However, in the face of a complex electromagnetic/radiation environment, the state of knowledge regarding this which impedes efforts to develop membrane shielding materials for future applications. With the continuous efforts by researchers, deeper insight was gradually being gained about the mechanism of shielding materials. Jin et al. [170] have proposed that a unique alternating multilayer structure could have an important role in shielding radiation. When the incident electromagnetic microwave (EMW) was eliminated, it was absorbed or dissipated in the material in the form of heat, and the internal transmission times are increased to form multiple reflections to weaken the EMW. In addition, an MXene layer provides a continuous thermal conduction network in the whole membrane, thus greatly enhancing the in-plane thermal conductivity of multilayer membrane materials (Figure 27a). Cheng et al. [171] have proposed that the inhomogeneity of a medium leads to multiple scattering phenomena and reflection of EMW in multi-channels. The macro sandwich cavity structure formed by the prepared AN@MXene/TW material greatly prolongs the transmission path of the electron beam, resulting in more absorption attenuation, which has better shielding performance than a single-sided coating (Figure 27b). Zhu et al. [172] have postulated that the electromagnetic synergistic network composed of reasonably designed conductive network and confined magnetic particles was the main reason why composite aerogels have excellent EMI performance while maintaining ultra-low reflectivity. The aligned layered structure of this type of aerogel delays the transmission of microwave by nearly infinite internal reflection and scattering, which provides space for effective attenuation of the electromagnetic cooperative network (Figure 27c). Shahzad et al. [173] had suggested that shielding was mainly attributable to multiple internal reflections generated by MXene structure. EMW can be reflected back and forth between layers (I, II, III, etc.) until it was completely absorbed by the structure (Figure 27d). Huang et al. [174] established a perfect double permeation structure to explain its mechanism (Figure 27e). On the one hand, the double-permeability structure produced more interfaces, and its resonance characteristics [175] absorbed numerous electromagnetic waves due to multiple reflections in the structure. On the other hand, the interaction between electric dipole and electromagnetic wave [176] strengthened the absorption of electromagnetic waves. Zhang et al. [175] have suggested that wave interference occured during the course of multiple reflections, and its possible resonance characteristics would promote the absorption of specific electromagnetic waves (Figure 27f). Wang et al. [177] prepared a shielding material with a layered structure of conductive pearls. When an electromagnetic wave reached the surfaces of the conductive pearls, it would interact with the carrier wave on the surface of MXene and thus become partially reflected. In addition, it would enter the inside of the conductive pearl layer, and the layered structure would reflect and scatter many times, resulting in the absorption and attenuation of electromagnetic energy. In addition, the existence of functional groups (-O, -OH, -F) and N atoms on the surface of MXene may lead to polarization under the action of an alternating electric field, resulting in polarization loss, which comprehensively enhanced the shielding effect (Figure 27g). Therefore, with the deepening of the research on shielding mechanism, the development of lightweight and efficient three-dimensional porous membrane shielding materials will become an important focus of future research.

## 6. Review of Composite Membrane Shielding Materials for Electromagnetic/Radiation Pollution

By combining different components, researchers have prepared a wide variety of composite materials with excellent electromagnetic/radiation shielding properties. The properties of some reported composite electromagnetic/radiation shielding materials are listed in Table 1. The SE value of most cellulose composite electromagnetic interference shielding materials was in the range of 25–70 dB in the frequency range of 8.2–12.4 GHz (X-band), and the SE values of some other membrane shielding materials can reach up to 91 dB. However, the properties of membrane shielding materials prepared with different composite materials are very different.

The thickness and density of a shielding material greatly affect its performance and applicability. Therefore, various parameters and properties, as well as the correlation among many factors and the performance response mechanism, should be comprehensively considered when developing membrane shielding materials. Cellulose was anticipated to have a very important role in the preparation of shielding materials because the world was turning toward sustainable and renewable materials, and cellulose and its derivatives have important characteristics such as biodegradability, biocompatibility, non-toxicity, high surface area, molecular polarization, switchable hydrogen bonding, and low cost [51,52,53,54]. The introduction of different types of nanomaterials improves the properties of cellulose and its derivatives. Combining a material such as cellulose with graphene or carbon nanotubes yields materials with good electromagnetic interference shielding performance, light weight, and low density. However, both graphene and carbon nanotubes are expensive, so the compromise between SE and cost should be considered when designing materials for practical applications. When cellulose is combined with metal or metal oxide, the resulting material has high electromagnetic interference SE, but its high weight and poor corrosion resistance limit its applicability. However, the EMI SE of the material prepared by combining cellulose with conductive polymer was lower than that of the above two materials. In addition, significant progress has been made through the combination of cellulose with MXene, and the obtained materials show excellent EMI shielding performance. Moreover, the use of 3D printing technology to design electromagnetic interference was a very promising preparation method, and combining theoretical simulation with experimental data will have an important role in optimizing manufacturing methods.

In addition, compared with dense thin membranes, three-dimensional porous materials can trap electromagnetic waves in pores and increase the number of reflections, which is beneficial to enhancing electromagnetic absorption loss. This is mainly due to the unique multi-reflection mechanism of porous structures, which can not only reduce the material’s density and improve the impedance matching characteristics of the absorbent, but also facilitate the adsorption, and recombination of powder or wave-absorbing nanomaterials, thus meeting the requirements of “thin, light, wide, and strong” electromagnetic shielding materials. However, in order to realize industrial application, it was necessary to find ways to further reduce the manufacturing costs and simplify the preparation methods. If low-density and ultra-light biomass porous carbon materials are used, it will be easier to construct binary, ternary, or even more composite absorbing materials and thus achieve stronger electromagnetic shielding performance, which will have broader applicability. However, to overcome the contradiction between impedance matching and attenuation characteristics and to achieve a synergistic enhancement of electromagnetic loss by organic coupling of various mechanisms, further research will be needed on the synergistic loss mechanism of multi-element three-dimensional porous composite absorbing materials. In addition, it was necessary to continue to investigate ways to construct multi-component composite three-dimensional porous electromagnetic shielding materials with stronger electromagnetic loss capabilities and a higher impedance matching level through microstructure design and to optimize the preparation processes leading to three-dimensional porous electromagnetic shielding materials. Finally, three-dimensional porous material-based electromagnetic shielding materials with high temperature resistance, corrosion resistance, compressibility, and flexibility can be developed to further improve the practicability and applicability of electromagnetic shielding materials.

In order to obtain a good shielding effect, it is necessary to modify filler materials via approaches such as morphology control, coating modification, and blending modification. Achieving this goal will enable the development of inexpensive new conductive fillers with high conductivity, good mechanical properties, and good compatibility with the shielding materials. If these types of fillers become available, it will be easier to form three-dimensional network frameworks, thereby improving the absorption rate of electromagnetic waves/rays and reducing the reflectivity and transmittance of membrane shielding materials. In addition, combined with advanced material characterization methods, various related factors that affect the shielding effectiveness of electromagnetic shielding materials are sorted out, the internal relationship of each influencing factor is revealed, and the electromagnetic shielding mechanism related to multiple factors in complex composite materials is analyzed. Therefore, it will be highly desirable to develop a new type of low-cost, and high-performance composite membrane shielding materials. The modification methods, the selection of modified materials and the preparation process all affect the performance of the composite membrane shielding materials together, and successfully taking these considerations into account will enable the construction of an efficient conductive network and a green shielding material that can be readily dispersed in its matrix. In particular, when designing composite membrane shielding materials, the shielding mechanism should be considered in a comprehensive manner.

## 7. Conclusions and Prospect

In summary, electromagnetic interference and radiation pollution seriously interfere with the normal work of precision electronic components and directly endanger information security as well as human health. Therefore, it is particularly important to develop efficient electromagnetic interference shielding materials to prevent the failure of high-precision electronic instruments and protect human health. The rapid pace of scientific and technological development has led to more demanding requirements for shielding materials, such as high absorption capacity, low density, wide frequency range, good thermal stability, good mechanical properties, light weight, flexibility, and low cost.

This paper mainly explores the recent progress that has been achieved with single structure design, multiple composite structure design, preparation technology, and the electromagnetic loss mechanism of membrane shielding materials designed to counteract electromagnetic/radiation pollution. It has been found that by introducing porous structures, constructing heterogeneous structures, designing multilayer structures, as well as utilizing 3D structures, simulations, fillers, and magnetic materials, the electromagnetic shielding performance of membrane shielding composites can be effectively improved. In addition, a shielding material can be endowed with other functional characteristics to realize the integration of structure and function. 

In the future, the development direction of membrane shielding materials will be mainly as follows:

(1) Simulation. WinXCOM, Auto-Zeff software, EGS software, MCNP software, CST software, COMSOL and other software programs are used for simulation so as to optimize the preparation process leading to membrane shielding materials. As computing technology continues to progress, the development of new and more accurate simulation software will likely provide further benefits in this area; (2) Multiple compounding of functional materials. The use of carbon-based functional materials such as conductive polymers and graphene or metals, fibers, or fabrics are promising ways to enhance the properties of shielding materials, and these strategies are likely to garner significant attention in the future; (3) Functional integration. With good shielding performance, multiple functions can be achieved, such as good wave absorbing performance, flame retardant performance, antibacterial performance, and radiation resistance; (4) Intelligence. By exhibiting a timely response to the surrounding environment, a smart or intelligent shielding material could adjust its internal structure and electromagnetic characteristics according to the changes in the surrounding environment; (5) Green and environmental protection. Researchers should make efforts to develop promising green electromagnetic interference shielding materials with low reflectivity and superior dynamic performance adjustment function that help reduce secondary electromagnetic pollution and can be applied in complex situations; (6) Self-repairing materials. The design of supramolecular networks to repair the damaged surface independently can be employed to impart membrane shielding materials with self-repairing capabilities, which can prolong the service lifetimes of these materials, reduce the costs associated with repairing or replacing the damaged equipment, and improve the safety for all users and the public.

In conclusion, optimizing the design of shielding systems from the perspective of theoretical simulation, developing new shielding materials with multiple functions, light weight, high efficiency, and high strength materials, and organically combining them to enhance the integration degree for the structure/function of the electromagnetic/radiation shielding systems are key aspects of current and future research in this field. Most importantly, researchers in the future should give full consideration to the unique properties of membrane shielding materials, such as porosity, multilayers, magnetism, and conductivity, which will enable the development of a wider range of applications for these materials (especially in harsh environments) and also help to address challenges encountered during production.

## Figures and Tables

**Table 1:** Summary of the properties of some membrane shielding materials.

Classification	Material	Frequency/GHz	SE/dB	Strength/MPa	Characteristics	Ref.
Metal	AgNWs/NC	8–12	60–76	-	High thermal and electrical conductivity	[97]
	AgNF					[178]
Polymer	EP/PES/MWCNT	3.94–12.4	23–90	2.55–69.7	Adjustable conductivity, good flexibility, production cost.	[28]
	PDMS/MWCNTs					[107]
	EVA@PDA@Ag					[175]
Concrete	WO_3_ and barit	0.122 (MeV)	99% (RPE)		Thermal durability and chemical corrosion resistance	[118]
Lead	PVA/pb(NO_3_)_2_	-	-	37.5	It has good attenuation characteristics for neutrons and γ rays.	[179]
Boron	BN/NFC	-	-	102	Good radiation resistance and neutron absorption performance.	[124]
3D	TiO2-Ti3C2Tx/rGO	30	58–65		Flexible, controllable and efficient	[126]
	Ti3C2Tx/(o-GNPs/PLA					[128]
MXene	AgNW@MXene/	8–12.4	44.96–58.4	11.7–422	Lightweight, strong flexibility and high shielding efficiency	[171]
	Ti3C2Tx/PANI/LM					[180]
	MXene/Epoxy					[177]
	MXene/GO					[181]
Carbon	PLA/PCL/8CNT/0.8IPU	9	35.6	-	Good electrical conductivity, light weight and stable chemical properties.	[175]
	CNTs/SBS					[182]
Fe	Fe3O4@CNT	8.2–12.5	30–91	0.1692–0.0432	Strong absorption and frequency bandwidth	[3]
	PS/TGO/Fe3O4					[172]
Cellulos	CNT-interface/cellulose	8.2–12.4	28–40	22.5	Thermal stability and easy processing	[159]
	Waste paper cellulose					[158]
Lead-free	Gd_2_O_3_/NR	Neutrons	-	8.29	High temperature resistance and oxidation resistance	[95]

## Data Availability

Not applicable.
